# Molecularly Imprinted Polymer Nanoparticles for Pharmaceutical Applications: Sample Preparation, Sensor-Based Detection, and Controlled Drug Release

**DOI:** 10.3390/polym17172283

**Published:** 2025-08-23

**Authors:** Sibel Büyüktiryaki

**Affiliations:** Department of Medical Services and Techniques, Yunus Emre Vocational School of Health Services, Anadolu University, Eskişehir 26470, Turkey; sbuyuktiryaki@gmail.com or sbuyuktiryaki@anadolu.edu.tr; Tel.: +90-222-3350580/3091

**Keywords:** molecularly imprinted nanoparticles, drugs, sample preparation, sensor, drug delivery, green synthesis, computational design, machine learning-assisted MIPs

## Abstract

Molecularly imprinted polymer nanoparticles (MIP NPs) are synthetic receptors with selective recognition sites for target molecules. They are employed instead of biorecognition elements in many applications due to their high affinity and selectivity, stability, easy preparation, and low cost. Their nanoscale size provides enhanced surface interactions, faster response times, improved biocompatibility, and effective cellular penetration, particularly in complex biological environments. MIP NPs provide high selectivity and structural versatility in the sample preparation, sensor-based detection, and controlled drug delivery, serving as promising alternatives to conventional methods. This review highlights the recent advancements in the synthesis and application of MIP NPs in three critical areas: sample preparation, sensor-based detection, and controlled drug release. Additionally, recent developments in green synthesis approaches, biocompatible materials, and surface functionalization strategies that are effective in the performance of MIP NPs are mentioned.

## 1. Introduction

Drugs play a significant role in the treatment and management of various diseases. An increasing variety of drugs is used to treat diseases in humans and animals. These drugs effectively relieve pain, extend life expectancy, and improve people’s quality of life. While the therapeutic uses of drugs are vital for human health, environmental contamination from these substances poses significant problems. Many sources, including the pharmaceutical industry, hospital waste, sewage disposal, drug misuse in animals and agriculture, and improper disposal of unused drugs, contribute to the release of these compounds into the environment. Even minimal concentrations of these substances in food, biological, and environmental samples can pose a substantial risk to human health [1,2]. The selective detection of drugs present in complex samples is essential to protect the environment and public health.

Drugs are mostly determined using gas chromatography (GC), high-performance liquid chromatography (HPLC), and combinations of these instruments with mass spectrometry (GC-MS, LC-MS) in routine analyses and research laboratories [3,4,5,6]. However, these devices are expensive and often require time-consuming sample pretreatment to detect low concentrations of drugs in complex samples, because their selectivity is insufficient. Although the analytical performance of these devices is quite good, the matrix effect limits the analysis of drugs at very low concentrations. Therefore, sample preparation is the most critical and time-consuming step in the analytical workflow. It is essential for the accurate analysis of trace amounts of drugs. Researchers are working to validate their methods by making sample preparation and determination of drugs faster, more sensitive, selective, and efficient.

Molecularly imprinted polymers (MIPs) are effectively used in various fields, including sample preparation [7,8], sensors [9,10,11], separation [12,13], controlled release [14,15], catalysis [16,17], cancer therapy [18,19], and imaging [20,21]. They simplify the time-consuming process of sample preparation thanks to their unique selectivity. In sample preparation, they provide excellent analyte enrichment in complex matrix environments. MIPs can be used many times in sample preparation processes, since they have very long-term stability [22]. In sensor systems, they serve as recognition elements, enabling selective, stable, and sensitive analysis. They are a powerful alternative to biorecognition elements because they can mimic the specific binding behavior of natural receptors. Although natural biorecognition elements have high specificity and binding capacity, they have disadvantages such as limited stability, high cost, and short shelf life. The superior stereospecific recognition capabilities of MIPs have made them attractive for use in biomedical applications [23].

In addition to their analytical uses, MIPs have also been effectively used in controlled drug delivery in recent years [14,24]. In these systems, a polymer matrix has a structure that allows drug molecules to localize to specifically defined binding sites and control their release. Molecular imprinting of the drug enables the formation of this selective polymer matrix. The targeted and controlled release of therapeutic agents can be achieved by releasing the target molecule from the polymer in response to a specific stimulus (e.g., pH, magnetic field, redox conditions, temperature) [18]. In these studies, parameters such as polymer composition, crosslinking density, selection of polymerization agents, and surface functionality affect the controlled release of the drug. Additionally, biocompatible and biodegradable components should be preferred to reduce the toxicity of the substances used.

Molecularly imprinted polymer nanoparticles (MIP NPs) combine the advantages of MIPs with the properties afforded by their nanoscale. The small size of MIP NPs allows more efficient interaction with target molecules, especially in complex environments. Thanks to their nanoscale, they exhibit faster response times, greater surface interaction, and more compatible behavior with biological systems compared to other MIPs [23,25,26]. Their small size ensures colloidal stability in biological environments and easier penetration into cells.

This article covers three major applications of nanoscale MIPs and evaluates recent advances in this field. To the best of my knowledge, no comprehensive study has addressed the wide range of applications of MIP NPs, such as sample preparation, sensors, and controlled drug delivery. Most reviews in the literature have discussed various applications of MIPs of different sizes. Notably, some studies mentioned nanoscale MIPs. For example, in 2020, Fresco-Cala et al. investigated the use of micro- and nano-sized MIPs in extraction and sensing applications [27]. In the study by Shevchenko et al., the scope was limited to nanoscale MIPs obtained through solid-phase synthesis for drug delivery [28]. Other synthesis methods were beyond the scope of their investigation. Similarly, Muhammad et al. investigated only the sensing and imaging applications of nanosized MIPs synthesized using the solid-phase synthesis method [29]. In contrast to previous studies, the present study highlights MIP NPs synthesized via emulsion, suspension, precipitation, sol-gel, electropolymerization, and solid phase synthesis, as well as emerging strategies, including green synthesis, computational design, and machine learning-assisted MIP NPs design.

This review provides a comprehensive examination of the pharmaceutical applications of MIP NPs under three main headings: sample preparation, sensor-based detection, and controlled drug delivery. The advantages and limitations of MIP NPs synthesized by various methods are discussed. This section also examines environmentally friendly synthesis approaches. It also addresses the use of computational and AI-assisted MIP designs to synthesize efficient and effective MIP NPs. Moreover, the effective utilization of MIP NPs in various extraction techniques has been examined, with particular emphasis on their recent achievements in pre-concentration and selectivity. The high selectivity of MIP NPs for drug enrichment from complex samples, as well as their contribution to analytical performance, is emphasized. It also highlights the potential use of MIP NPs as recognition elements in sensor systems. In particular, electrochemical and optical sensors have demonstrated the reliable and sensitive detection of low concentrations of drugs thanks to the selective binding sites of these nanoparticles, which mimic natural biorecognition systems. As a third application, the potential of MIP NPs in controlled drug delivery systems is evaluated. Recent studies are also discussed to present current advancements and emerging trends in the field.

## 2. MIP NPs Synthesis Methods

Molecular recognition is a fundamental biological mechanism that drives life. It plays a key role in various biological activities, including interactions such as antibody-antigen recognition, enzymatic catalysis, and signal transduction. Fischer first introduced the concept of molecular interactions in 1894, presenting it with his “lock-key” model. This model serves as the basis for enzyme-substrate interactions. This concept states that complex formation occurs as a result of intermolecular interactions between complementary functional groups on the lock or enzyme and the desired key or substrate (analyte) [30,31]. Pauling’s theory on the formation of antibodies inspired the development of the molecular imprinting technology [32,33]. Natural recognition elements, including antibodies, enzymes, nucleic acids, and cells, utilize noncovalent interactions such as hydrophobic interactions, ionic bonds, van der Waals forces, and hydrogen bonds to bind to their targets with remarkably high affinity. The limited long-term chemical and physical stability of natural recognition elements, along with their relatively high cost and the need for skilled labor, has motivated scientists to explore alternative methods that can reduce these drawbacks. The molecular imprinting method represents an important application area of this concept [34,35].

**Figure 1:**
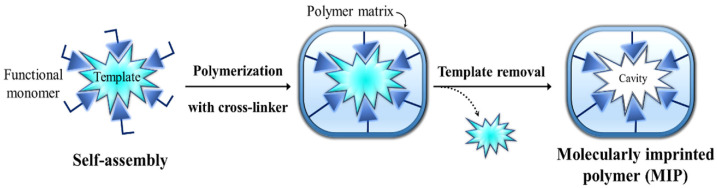
The schematic representation of MIP preparation (Reprinted with permission from [11]).

The MIP technique is based on the complexation of a template molecule with a functional monomer(s) in a suitable solvent through non-covalent or covalent interactions (Figure 1). After polymerization, the template molecule is removed, and cross-linking creates binding sites specific to the target molecule. These binding sites are compatible with the template molecule’s functional groups, size, shape, and position. Essentially, a molecular memory polymer is created that can selectively bind to the template molecule. MIPs are produced to mimic biological receptors and can bind to template molecules with similar affinity and specificity compared to their natural counterparts [35,36]. They have longer stability, shelf life, and reusability than natural recognition systems [22]. The technique is simple and inexpensive. It is also more resistant to degradation under harsh environmental conditions, such as acids and bases, high temperatures, and pressures, than its biological counterparts. Additionally, the synthetic design in the molecular imprinting technique provides suitable interaction spaces for the target molecule, even though there is no natural receptor [9].

### 2.1. Precipitation Polymerization

Precipitation polymerization is a type of polymerization in which the monomer, cross-linker, template molecule, and initiator are soluble in the reaction medium, but the resulting polymer is not. This method enables the formation of uniformly shaped and sized polymers, resulting in reproducible results. The high porosity of the polymer improves its binding capacity. However, using large volumes of solvent can weaken the interactions between the template and the monomer [37]. Wang et al. successfully synthesized fluorescent MIP NPs for the detection of paracetamol using a one-step precipitation method, which involved polyvinylcarbazole, poly(styrene-co-maleic anhydride), and paracetamol [38]. This approach facilitates the preparation of uniformly sized MIP NPs without complicated procedures or extra additives, making it a practical and cost-effective polymerization method.

### 2.2. Emulsion Polymerization

Emulsion polymerization enables the production of high molecular weight polymers with uniform size in a short time. Emulsion conditions and the amount of surfactant also affect particle size. Removing surfactants used in emulsion stabilization from the polymer is very challenging. Since surfactants are toxic to cells, their use is limited in biological applications [39]. Tang and colleagues successfully detected olaquindox residue in animal products using uniformly sized magnetic MIP NPs prepared through emulsion polymerization [40]. Olaquindox and its main metabolite, methyl-3-quinoxaline-2-carboxylic acid, were determined selectively, sensitively, and simultaneously.

### 2.3. Sol-Gel Polymerization

Sol-gel polymerization, which involves the hydrolysis and condensation of alkoxides in acidic or basic media, yields a biocompatible and non-toxic polymer. The polymer can be easily prepared without requiring high temperatures. Eco-friendly solvents, such as water and ethanol, can be utilized for polymer preparation [41,42]. The Cu^2+^ mediated magnetic MIP NPs (CM-mMIP) were synthesized by He via the sol-gel method and used for selective enrichment and determination of trace amounts of captopril in rat plasma [43]. They provided the pre-organization step by forming coordination complexes of captopril with Cu^2+^ ions and the functional monomer. After the prepared pre-complex interacted with Fe_3_O_4_ nanoparticles, it was polymerized using tetraethyl orthosilicate (TEOS) as a crosslinker in an acidic medium. They achieved high adsorption capacity and quite good enrichment factor by coating the Fe_3_O_4_ core with a 3–5 nm thick MIP layer.

### 2.4. Surface Imprinting

In the surface imprinting method, polymerization occurs on the surface of solid supports such as carbon nanotubes (CNTs), Fe_3_O_4_, SiO_2_, metal-organic frameworks (MOFs), and carbon dots. Since the recognition sites are located on the surface, the binding and removal of the target molecule occur more rapidly compared to other imprinting methods [44,45]. This approach is particularly favored for the recognition of large target molecules, such as proteins. It provides a significant advantage in real-time detection platforms by combining selectivity with fast response time.

### 2.5. Electropolymerization

Electropolymerization is a simple and rapid method frequently used in sensor applications. Polymer films are obtained by applying a potential to the electrode surface using monomers such as pyrrole, aniline, o-phenylenediamine, thiophene, 3,4-ethylenedioxythiophene (EDOT), and dopamine. Direct electrochemical energy is used instead of free radical initiators, eliminating the need for harsh conditions such as heat, pressure, or an inert atmosphere [46]. Homogeneous and thin MIP films ranging from nm to µm thickness can be produced. These polymers provide high selectivity and sensitivity for the recognition of target analytes. However, removing the target molecule from the polymer is challenging, and the number of monomers is limited. In the study of Zhang et al., a MIP film was fabricated via electropolymerization on a CoWO_4_/g-C_3_N_4_/glassy carbon electrode using cyclic voltammetry (CV) in the presence of furazolidone (a nitrofuran antibiotic) and o-phenylenediamine [47]. The resulting MIP/CoWO_4_/g-C_3_N_4_ composite simultaneously provided a dual-functional system, enabling photocatalytic degradation of furazolidone and the selective detection of furazolidone.

### 2.6. Solid-Phase Imprinting

Piletsky and his team, pioneers of the solid-phase imprinting technique, immobilized target molecules (melamine, vancomycin, and a peptide) onto the surface of glass beads using covalent bonds before polymerization [48]. Polymerization was then successfully carried out under UV light, at low temperature, and within a short reaction time. Following this process, high-affinity MIP NPs (called nanoMIPs) were obtained through temperature-controlled elution. Binding the target molecule to the solid support before polymerization improves the homogeneity of the binding sites and enables the formation of highly specific nanoMIPs. The synthesized nanoMIPs demonstrated strong affinity for their target molecules. The developed synthesis method is suitable for automation. It is faster than other methods and requires less solvent, providing environmental and economic benefits. However, this technique has some limitations. The overall yield is relatively low, and interactions between amino groups can lead to the formation of multilayer silane structures on the substrate surface. To prevent template leakage, loosely bound layers must be removed by washing before imprinting. Additionally, free amines that are not covalently bound to the template molecule may interfere with polymerization by decomposing the persulfate initiator. As an alternative, iodo-terminated silanes or thiol-containing molecules have been proposed for template immobilization [29]. The low stability of the silane layer to water is another limitation. Therefore, dipodal silanes were used to increase the surface hydrophobicity, thus improving both template immobilization and nanoMIPs yield.

To determine the effect of polymerization time on binding properties, Chiarello and coworkers synthesized ciprofloxacin-specific nanoMIPs using a solid-phase imprinting technique [49]. They produced nanoMIPs with high binding affinity but low selectivity in a short polymerization time (15 min). Polymerization times ranging from 30 min to 2 h resulted in nanoMIPs with both strong binding capacity and high target selectivity. In contrast, prolonged polymerization (>2 h) led to nanoparticles with reduced affinity, faster dissociation kinetics, and limited selectivity. In the early stages of the polymerization process, the flexible nature of the polymer chains allows them to conform quickly to the template, resulting in high binding affinity but low selectivity. As polymerization time increases, the nanoMIPs become larger and more structurally complex. The binding sites become more prominent but less accessible. This causes a decrease in binding affinity and rate.

### 2.7. Green Strategies for MIP Synthesis

Researchers have recently turned to MIP synthesis following green chemistry principles to reduce environmental impacts and minimize the use of toxic components. This approach favors more sustainable and environmentally friendly alternatives to the toxic solvents, initiators, or monomers used in traditional methods. Recently, the use of chitosan, cellulose, cyclodextrin, itaconic acid, and ionic liquids as monomers has become widespread. Ionic liquids (ILs), supercritical CO_2_, deep eutectic solvents (DESs), ethanol, and isopropanol are preferred as green solvents. Additionally, using a dummy template instead of a toxic target molecule is also possible. Energy consumption can be reduced by achieving polymerization in a shorter time using UV, ultrasound, or microwave-assisted methods [50,51,52,53]. A tool called AGREEMIP was recently developed to evaluate and compare MIP synthesis methods in terms of environmental sustainability [54]. AGREEMIP is based on 12 separate assessment criteria that consider various parameters related to MIP syntheses, such as solution composition, energy use, and experimental conditions. The scores obtained from each criterion are processed according to predetermined weights, resulting in an overall score ranging from 0 to 1. This assessment can be performed using open-source, user-friendly software that can be downloaded from mostwiedzy.pl/agreemip.

### 2.8. Computational MIP Design

Computational MIP design focuses on optimizing experimental parameters, including the selection of monomer, crosslinker, template, and solvent, as well as their respective molar ratios [55,56]. The calculation of binding energies, hydrogen bond sites, and intermolecular electrostatic interactions enables the identification of the most stable monomer-template complex. The most stable complex formed by the template and functional monomer is selected based on the strongest interaction [57]. Because computational MIP design addresses various aspects of the molecular imprinting process, it enables the synthesis of MIPs with better selectivity and sensitivity. It also reduces the workload of the experimental process by optimizing the parameters used. Optimization can be performed using atomic-level simulations (molecular dynamics, Monte Carlo, molecular mechanics) or quantum mechanics level simulations (Density Functional Theory, Hartree-Fock, semi-empirical). The Hartree-Fock (HF) method provides high accuracy and good agreement with experimental data. The Monte Carlo method is more robust in terms of assumptions, but is difficult to implement for complex samples and does not apply to electronic properties. The molecular mechanics (MM) method is fast and simple. But it does not apply to static bonding and electronic properties. Mohsenzadeh et al. reported that combined quantum mechanical/atomistic approaches provide greater flexibility and versatility in modeling large systems [58].

Density Functional Theory (DFT) is extensively applied in many studies due to its favorable balance between accuracy and computational cost. In the computational stage of MIP design, Çetinkaya and colleagues used quantum chemical calculations to investigate interactions between ribavirin and selected functional monomers (p-aminophenyl boronic acid (p-APBA), uracil methacrylate (UraM), pyrole, 2-hydroxyethyl methacrylate) [59]. They calculated the fundamental properties of molecules by applying the hybrid density functional theory (DFT/B3LYP) approach using the Gaussian 2016 program package. For this purpose, optimization of monomer-template complexes was performed, and binding energies were calculated. From the electrostatic potential charge distribution maps on the molecule surface, it was shown that the molecules were polar and thus could form hydrogen bonds or non-covalent bonds. They also used BIOVIA Discovery Studio 2022 and AutoDock programs for molecular docking studies to predict interactions between ribavirin and functional monomers. The obtained results guided the selection of the most stable complex and the determination of the most suitable monomer for experimental MIP synthesis. Docking calculations for the ribavirin-p-APBA pair indicated that a maximum of five pyrole molecules could be bound to the structure, in agreement with experimental findings. The optimum ratio for the ribavirin-UraM complex was calculated as 1:1, which was also confirmed by experimental results. The same research group successfully determined the optimal monomer-to-template ratio for the detection of camostat mesylate using a similar approach [60]. These findings demonstrate the critical role of computational modeling in MIP design. The use of computational modeling in MIP design reduces trial-and-error steps by enabling the selection of optimal conditions before synthesis.

Damayantin’s group used the DFT method, GFN2-xTB, and molecular dynamics simulation to predict the optimal functional monomer, solvent, and stoichiometric ratio for remdesivir imprinting [61]. The solvent screening was performed using the GFN2-xTB method. Acetonitrile provided the lowest binding energy (ΔE) and Gibbs free energy change (ΔG) among the solvents tested. Based on the molecular dynamics simulation results, the radial distribution function (RDF) value was calculated. The RDF value describes the probability distribution of finding the functional monomer at a given distance from the template. The target molecule/functional monomer complex with a 1:1 ratio exhibited the highest RDF value. The association constant was calculated as 1825.8 M^−1^ using the Benesi-Hildebrand equation. Experimental results, supported by association constant evaluation and Job’s plot analysis, indicated that the complex with a 1:1 ratio exhibited the most favorable complex formation. In another study by the same group [62], 2-acrylamido-1-ethanesulfonic acid was determined as the most suitable monomer for preparing fluconazole-imprinted polymers, based on its binding affinity, hydrogen bonding, complexation energy, and thermodynamic properties. Frontier molecular orbital analysis revealed that chloroform was the most suitable solvent for complex formation.

In recent years, the number of studies using methods such as quantum chemical calculations, molecular docking, and molecular dynamics simulations has increased significantly. These methods can predict optimal template-monomer interactions and enable the demonstration of binding mechanisms that are difficult to detect experimentally. Computational MIP design enables the determination of the optimal monomer/template ratio, improves the performance of synthesized MIP particles, and significantly reduces experimental workload. Among different functional monomers, the functional monomer that interacts most strongly and selectively with the target molecule can be identified. Computational strategies should be more widely used in MIP research due to their efficiency, cost-effectiveness, and performance-enhancing advantages. Incorporating these methods into the MIP design process will accelerate the development of application-oriented MIPs and facilitate broader use in analytical, biomedical, and environmental applications. Therefore, it is anticipated that these emerging approaches will be widely used in MIP design.

### 2.9. Machine Learning Assisted MIPs

Artificial intelligence (AI) has been widely applied in various fields, including health, engineering, materials science, pharmaceutical sciences, and chemistry, in recent years. It can process large datasets and recognize relationships. It can use this information to make predictions, classifications, and recommendations [63,64]. A review by Karasu and colleagues, published in 2024, comprehensively examined the opportunities that could arise from the combination of AI and MIPs [65]. They evaluated the algorithms used in this approach. They also emphasize that more efficient and targeted approaches can be implemented by deriving meaningful results from big data. The authors emphasized that the widespread use of AI in MIPs in the future could lead to significant advances in many applications, from materials science to biosensors.

In 2021, Lowdon and colleagues investigated the potential of machine learning (ML) algorithms to simulate the binding affinities of MIPs. In this study, the multitask regressor model and the graph convolution model provided the best performance in predicting binding affinities. While the models performed well during training, they performed poorly during testing due to the limited dataset. Furthermore, the calculations used data from heterogeneous polymers obtained through bulk polymerization, making learning by computational methods difficult. A more homogeneous MIP synthesis could be chosen, which would be easier for ML algorithms to learn and predict.

Yarahmadi and colleagues developed an approach to predict riboflavin absorbance using ML algorithms such as gradient boosting (GB), Ada boosting (AdaBoost), extra trees, and random forest algorithms [66]. They investigated template molar ratio, monomer molar ratio, crosslinker molar ratio, loading time, stirring speed, and pH to optimize the polymer synthesis conditions. Among the algorithms used, the GB and AdaBoost algorithms showed high performance in predicting riboflavin absorption. They found the mean absolute error and mean square error of the GB model as −0.003711 and −0.000078, respectively. Developing a model that can accurately predict riboflavin absorption is crucial. Using ML to predict riboflavin absorbance is a creative solution to overcome the challenges of optimizing polymer synthesis conditions. Researchers aim to add the outputs of a single variable and predict the results of different analytical instruments. Integrating ML into the polymer synthesis process will pave the way for significant advances in the design of more advanced and high-performance polymers in the future. The approach used in this study may reduce workload.

Kundu and colleagues prepared four different MIP-QCM sensors to classify lemongrass essential oils according to their citral concentration [67]. The developed sensors demonstrated excellent repeatability and reproducibility. Principal component analysis successfully clustered the four sample types, achieving a high separability index of 6.514. They also achieved the best performance (Precision: 92.30%, Accuracy: 85.687%) from the k-nearest neighbor model. Partial least squares regression gave an overall prediction accuracy of 96.64%. These chemometric methods demonstrate that combining QCM with MIPs is a highly promising approach. Additionally, this approach could be used to identify other molecules in the future.

In another interesting study, Zhang et al. synthesized four different fluorescence CDs-MIP sensors to distinguish twelve different sulfonamides simultaneously [68]. Data from these sensor arrays were analyzed and trained using nine different machine learning algorithms. Among them, linear discriminant analysis achieved the highest classification accuracy, reaching 98.96% accuracy in identifying 480 concentration-independent samples. This method also enabled the simultaneous determination of the twelve sulfonamides, with a detection limit as low as 0.026 µg/mL. This algorithm showed high potential in terms of accuracy and precision, ensuring the correct classification of data. This study provides an important strategy for screening sulfonamides and can be extended to the simultaneous recognition of other structurally similar compounds. Simultaneous multi-component SA screening is highly promising.

AI is particularly useful in modeling and optimizing complex systems. It can reduce costs and increase efficiency by focusing on experimental processes. However, the success of this technology depends on the algorithms used and the quality of the datasets. Homogeneous and reliable data are essential for producing accurate predictions. In molecular imprinting design, analysis of large datasets is expected to offer significant potential for determining material composition, classifying the resulting data, and enhancing decision-making [65]. Processing large-scale experimental datasets will enable the identification of relationships that are difficult to detect with traditional computational methods. It can be used to predict the functionality of MIPs before experiments by determining the optimal interaction between the target molecule and the functional monomer. It can also be used to optimize factors such as reaction temperature, the amount of monomer, crosslinker, initiator, and solvent, which are highly influential in polymer performance. To effectively use AI systems in molecular imprinting, researchers need expertise in both AI and chemistry. As they improve in this area, they will be able to use it more effectively in MIP design.

## 3. Applications of MIP Nanoparticles

### 3.1. Sample Preparation

Solid phase extraction (SPE) is one of the sample preparation techniques used to separate and concentrate target compounds from complex samples. In molecularly imprinted solid phase extraction (MISPE), MIPs are used instead of traditional adsorbents. MISPE stands out as a more selective type of SPE. Traditional adsorbents often have limited selectivity due to their non-specific interactions. MIPs have highly selective binding sites that recognize the size, shape, and functional groups of the target analyte, thus increasing both the selectivity and the efficiency of the extraction process, especially in complex samples [69,70]. While conventional adsorbents typically suffer from limited reusability, MIPs exhibit superior reusability, making them more advantageous in repeated extraction applications.

The MISPE technique can be applied in either an online or offline format. In the online MISPE approach, the MIP phase is directly integrated as a column into systems such as high-performance liquid chromatography (HPLC) or liquid chromatography mass spectrometry (LC-MS). In the offline approach, the MISPE cartridge is used separately during the sample preparation. The conditioning, sample, washing, and elution solutions are sequentially passed through the MIP sorbent. The resulting analyte solution is then analyzed. This approach is useful in method development and offers compatibility with various analytical instruments [71].

Cheng and colleagues developed an MISPE column for the selective detection of ceftiofur sodium (an antibiotic) that can be found as a veterinary drug residue in milk and animal tissues [72]. In preparing the MIP NPs, poly(styrene-divinylbenzene) (PS-DVB) was used as a carrier, N, N′-methylene bisacrylamide (MBA) as a cross-linker, and azobisisobutyronitrile (AIBN) as an initiator. 1-allyl-3-vinylimidazolium bromide and 2-acrylamide-2-methylpropanesulfonic acid (AMPS) were employed as functional monomers. Maximum adsorption was achieved with a sample volume of 10 mL at pH 7.0. Desorption was performed using 1.5 mL of a methanol-water mixture (70:30, *v*/*v*). The prepared MISPE column also showed outstanding selectivity for the target compound ceftiofur sodium in the presence of other compounds such as metronidazole, ceftriaxone sodium, and amoxicillin sodium. The limit of detection (LOD) was 0.0015 mg/L, and recovery rates ranged from 91.9% to 106.8% across milk, chicken, pork, and beef samples. After 20 extraction cycles, the recovery rate of the MISPE column for ceftiofur sodium remained above 95%, with no degradation of the MIP NPs observed.

Liu et al. developed a ZIF-67-based molecularly imprinted nanocomposite for selectively extracting non-steroidal anti-inflammatory drugs (NSAIDs) from environmental water and soil samples [73]. 1-allyl-3-vinylimidazolium tetrafluoroborate was used as the functional monomer. The maximum adsorption capacities of the developed ZIF-67-MIP nanocomposite for diclofenac sodium, flunixin meglumine, and nimesulide were 88.70 mg/g, 79.78 mg/g, and 19.24 mg/g, respectively. The results showed that the developed ZIF-67-MIP adsorbent exhibited notable selectivity towards the target compound diclofenac sodium. They achieved excellent recovery results in soil and water samples using the MOF-MISPE column. It was also reported that the nanocomposite maintained high adsorption capacity over 45 cycles.

Solid phase microextraction (SPME), developed by Pawliszyn and Arthur, is a simple, rapid, sensitive, and environmentally friendly sample preparation technique [74]. It was designed to overcome the drawbacks of conventional extraction techniques, simplify sample preparation steps, and enhance analytical efficiency [75].

Haghgouei and Alizadeh successfully developed a flexible, paper-based, electrochemically supported solid-phase microextraction (EC-SPME) technique for selective detection of naproxen, an NSAID, in human serum [76]. Initially, they formed a conductive layer on the paper surface through chemical polymerization using pyrrole, ammonium persulfate (APS), hydrogen peroxide (H_2_O_2_), and various anionic dopants. Then, they prepared a nanoscale conductive MIP film on the paper surface via electrochemical polymerization with CV in the presence of pyrrole and the target analyte. The prepared paper-based EC-SPME sorbent displayed excellent selectivity toward naproxen in the presence of other competing compounds such as ibuprofen, diclofenac, mefenamic acid, and salicylic acid. High recovery rates, from 90% to 103%, were observed in human serum samples. It was reported that the flexible, paper-based conductive MIP material retained its performance even after 55 consecutive extraction-desorption cycles.

In-tube solid-phase microextraction (IT-SPME) is an automated sample preparation technique used for extracting target analytes from liquid samples. It is an environmentally friendly method that reduces the need for solvents. IT-SPME consists of an adsorbent placed in a hollow, closed capillary column. MIPs are placed in a closed capillary column and are widely used as adsorbent materials due to their high selectivity. Due to their stable structure, MIPs can be used in many extraction cycles [77].

Kefayati et al. combined an online IT-SPME method with HPLC-UV analysis for the selective extraction of carbamazepine from urine and plasma samples [78]. First, they synthesized copper oxide (CuO) nanosheets on the inner surface of the copper tube using the chemical method. Then, they coated the CuO surface with carbamazepine imprinted polypyrrole polymer (MIP@CuO) using the in situ electrodeposition method. The linear range for carbamazepine was determined as 0.05–500 µg/L in water samples and 0.10–500 µg/L in urine and plasma samples. The obtained results confirmed that the MIP-based adsorbent showed excellent recognition ability and selectivity towards carbamazepine in the presence of diclofenac, ibuprofen, and fenofibrate. The prepared MIP-based adsorbent showed high mechanical resistance, chemical stability, and reusability (50 extraction cycles). IT-SPME is a reliable technique for repeatable analyses that is compatible with automated systems.

Dispersive solid-phase microextraction (DSPME) is a modern and efficient sample preparation technique in which a small amount of solid adsorbent is directly dispersed into the sample containing the target analyte. Since the adsorbent interacts directly with the target molecule, it provides significant improvements in extraction capacity and processing time. The adsorbent is separated using filtration or centrifugation, and then the analyte is desorbed from the solid phase. The DSPME technique is a faster, more environmentally friendly, and economical technique compared to classical extraction techniques. This technique requires less solvent or adsorbent. Extraction efficiency can be increased using a vortex or an ultrasonic bath. MIP adsorbents provide additional advantages such as high selectivity, extraction efficiency, and reusability when used in this technique [79,80,81].

**Figure 2:**
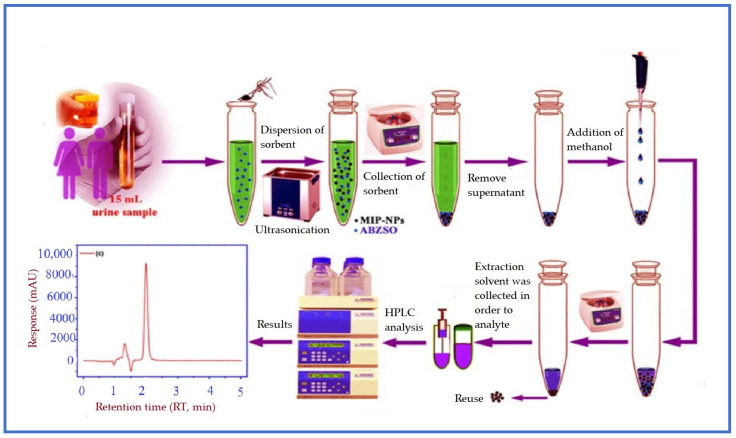
Schematic representation of the DSPME steps and detection of albendazole sulfoxide (Reprinted with permission from [82]).

Alipanahpour Dil and colleagues successfully prepared the albendazole sulfoxide-imprinted MIP NPs for the selective determination of albendazole sulfoxide, an anti-worm drug, in human plasma, urine, and water samples [82]. The use of the prepared MIP NPs in the DSPME technique is schematized in Figure 2. The extraction procedure was optimized using a central composite design to determine the ideal extraction conditions. They found a wide linear range (0.4–4200 ng/mL), low detection limit (LOD: 0.074 ng/mL), and satisfactory recovery values (95.7–103.0%). The binding capacity of the prepared MIP-based adsorbent was found as 63.69 mg/g. In addition, the imprinting factor of this adsorbent for albendazole sulfoxide was found to be 2.363, indicating that MIP NPs are highly selective over NIP NPs. Selectivity studies of the MIP NPs were performed with competitive compounds such as mebendazole, oxibendazole, triclabendazole, and tiabendazole and were found to be highly selective towards albendazole sulfoxide. The advantages of this method are that the synthesized MIP NPs can be reused up to eight times, and effective desorption can be achieved using a small amount of organic solvent. DSPME is an effective technique that can be preferred for low-cost, environmentally friendly, and rapid analyses.

In the magnetic solid-phase microextraction technique (MSPME), the solid phase can be easily separated from the liquid medium within seconds using an external magnetic field, without the need for time-consuming procedures such as filtration or centrifugation. The technique is rapid and straightforward, even for large sample volumes. The MSPME technique can also be adapted to dispersive formats. MSPME offers additional advantages for the determination of low-concentration analytes, since it requires smaller amounts of sample, solvent, and adsorbent. Nanostructured magnetic adsorbents have a large surface area to bind analytes, resulting in increased extraction capacity [83,84,85]. Metal oxides such as γ-Fe_2_O_3_ (maghemite), α-Fe_2_O_3_ (hematite), Fe_3_O_4_ (magnetite), and NiO, magnetic metals such as Mn, Fe, Co, and Ni, and spinel ferrites such as MgFe_2_O_4_, CoFe_2_O_4_, and MnFe_2_O_4_ can be used as magnetic cores [86]. These cores are usually coated with a SiO_2_ layer using tetraethyl orthosilicate (TEOS), 3-aminopropyltrimethoxysilane (APTMS), or 3-aminopropyltriethoxysilane (APTES) to prevent aggregation and oxidation of the magnetic core. A stable MIP layer is formed on the surface of magnetic nanoparticles via free radical polymerization using vinyl groups such as 3-(methacryloxy) propyl trimethoxy silane (MPS) [87], (3-acryloyloxypropyl) trimethoxy silane, vinyltrimethoxysilane (VTMS), and 3-vinyltriethoxysilane (VTES) [88]. The use of magnetic MIP NPs in a core-shell structure as adsorbents provides advantages such as extraction efficiency, enrichment factor, selectivity, and reusability [50]. The MSPME technique offers significant advantages in analytical chemistry with its rapid phase separation, fast adsorption-desorption kinetics, and ease of operation.

**Figure 3:**
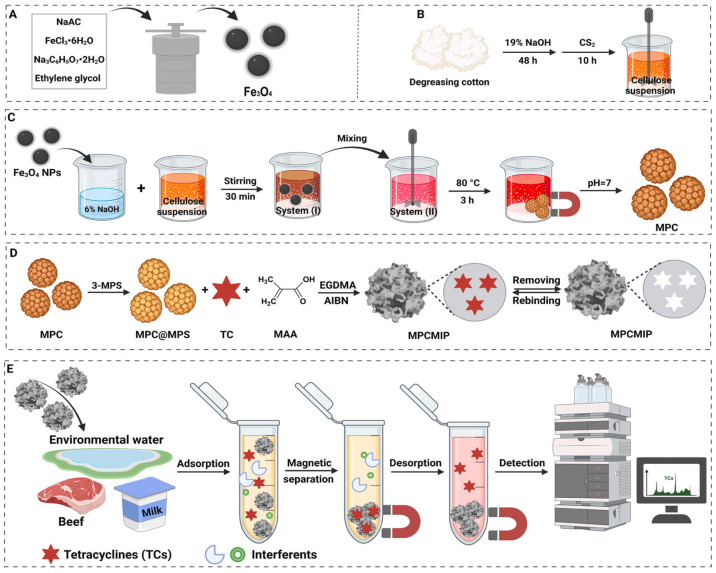
(**A**) Synthesis of magnetic nanoparticle by solvothermal method, (**B**) Preparation of cellulose suspension from cotton, (**C**) Preparation of magnetic porous cellulose, (**D**) Preparation of tetracycline imprinted magnetic nanoadsorbent, (**E**) Schematic representation of the use of magnetic MIP-based adsorbent in dispersed magnetic solid phase microextraction (DMSPME). (Reprinted with permission from [89]).

Li and co-workers synthesized a magnetic molecularly imprinted adsorbent that can selectively recognize and separate tetracycline antibiotic from environmental water and food [89]. They synthesized Fe_3_O_4_ nanoparticles via the solvothermal method (Figure 3A), and cellulose suspension was obtained from cotton (Figure 3B). Magnetic porous cellulose (MPC) was synthesized by dispersing an Fe_3_O_4_-cellulose aqueous mixture into an oil phase composed of Span 60 and potassium oleate, and subsequently reacting the emulsion at 80 °C (Figure 3C). The obtained MPC was functionalized with MPS and then polymerized in the presence of methacrylic acid, AIBN, ethylene glycol dimethacrylate (EGDMA), and tetracycline (Figure 3D). Tetracycline was desorbed from the polymer using a methanol-acetic acid solution. The prepared magnetic MIP-based adsorbent was used in dispersed magnetic solid-phase microextraction (DMSPME) of tetracycline from water and food (Figure 3E). In this study, the adsorption capacities of MIP NPs-based adsorbent for tetracycline, demeclocycline, and minocycline were determined to be 14.5, 10.1, and 11.0 mg/g, respectively. Reusability studies confirmed that the prepared magnetic MIP adsorbent could be used four times without a significant loss in recognition efficiency. Additionally, rapid phase separation was achieved using a magnet, without the need for filtration or centrifugation. The adsorbent also exhibited high selectivity toward tetracycline compared to structurally similar molecules, including oxytetracycline, chlortetracycline, and doxycycline.

Fathi and colleagues developed a new magnetic nanocomposite (MIP@COF@MIL-156@Fe_3_O_4_) for the selective extraction of montelukast (a drug used in the treatment of asthma and allergic rhinitis) from human plasma and urine using dispersed magnetic solid phase microextraction (DMSPME) [90]. For the adsorption step, 2 mg of MIP-based sorbent was added to the sample and vortexed for 8 min at pH 8.0. The target molecule was desorbed using 250 µL of acetonitrile for 2 min, then the sorbent was removed with a magnet, and the solution was analyzed using LC/MS-MS. The synthesized MIP-based nanocomposite was found to be highly selective towards the target montelukast compared to zafirlukast and zileuton. The detection limit of montelukast from urine was achieved as 0.04 ng/mL. The developed DMSPME method is suitable and reusable for the selective analysis of montelukast in biological samples.

A different study by Sabbaghi et al. investigated the application of MIP-based DMSPME for selectively detecting glibenclamide (an oral anti-diabetic drug) in wastewater, human urine, and plasma samples [91]. The MIP shell was synthesized through precipitation polymerization on functionalized magnetic iron nanoparticles, using EGDMA as the cross-linker and allylamine and itaconic acid as the functional monomers. Factors such as magnetic MIP mass, eluent type, eluent volume, adsorption time, desorption time, pH, temperature, ionic strength, and sample volume were optimized to achieve an effective adsorption-desorption process. Glibenclamide was effectively desorbed from the adsorbent using 500 µL of a methanol and acetic acid mixture (8:2 *v*/*v*) in an ultrasonic bath for 5 min. The detection limits of glibenclamide using HPLC-DAD and UV-vis spectrometry instruments were found to be 0.3 µg/L and 4.0 µg/L, respectively. Other antidiabetic drugs such as pioglitazone, glipizide, and rosiglitazone were also analyzed to evaluate the specificity and selectivity of the prepared sorbent. The recovery values for all real samples were found to be in the range of 89.4% to 102.9%. Its short application time, low solvent consumption, and reusability of the adsorbent offer cost-effective advantages.

The thin-film microextraction (TFME) technique offers several advantages, including fast mass transfer kinetics and high extraction capacity [92]. MIPs are suitable for use in this extraction technique. In an important study performed by Mirzajani and Kha, a new TFME technique was developed to selectively determine the antibacterial sulfonamides in milk, eggs, and chicken meat [93]. Nanocomposite thin films were prepared via electrospinning by combining a nanocomposite containing a bimetallic MOF, halloysite nanotubes, and a DES with sulfadiazine-imprinted polymer and polyacrylonitrile solutions. The sulfadiazine imprinted thin film was placed in a syringe and interacted with the sample solution to selectively bind the target molecules to the film surface. This adsorption process was optimized according to the Box-Behnken design, and the thin film was interacted with the sample solution in an ultrasonic bath for 9 min. The obtained limits of detection and limit of quantification ranged from 0.003 to 0.004 µg/L and 0.011 to 0.013 µg/L, respectively. The recovery values were found to be in the range of 92% to 101%. These results revealed that the developed nanocomposite film is an effective sorbent for the rapid and accurate extraction of sulfonamides that may be harmful to food safety.

Table 1 presents various applications of MIP NPs-based extraction techniques for the selective extraction of different drug classes, including antibiotics, an antiepileptic drug, an antidepressant drug, anticancer drugs, and an analgesic drug.

In another study employing the TFME technique, a tramadol-imprinted polymer was synthesized using chitosan, a biocompatible and natural biopolymer, as an environmentally friendly approach [107]. Tramadol is a narcotic central analgesic with abuse potential. The synthesized MIP NPs-based adsorbent was effectively used for the selective extraction of tramadol from saliva, urine, and wastewater samples. Desorption was effectively accomplished within ten minutes using only a minimal volume (400 µL) of acetone. In this study, the recovery values of the tramadol were in the range between 80.7% and 106.0% for real samples. The determination of tramadol was performed quickly and practically via smartphone-based colorimetric analysis. The imprinting factor of the prepared MIP-based thin film was found to be 13.99, indicating an excellent specific binding capacity of the MIP-based thin film towards the non-imprinted polymer (NIP). This method provides a sensitive, selective, and environmentally friendly alternative approach for the detection of tramadol.

Stir bar sorptive extraction (SBSE) is a sample preparation technique that enables the preconcentration of analytes by coating the outer surface of the magnetic stir bar with an adsorbent. This stir bar is placed directly into the liquid sample and stirred for a certain time. During this time, analytes transfer from the liquid phase to the adsorbent surface via sorption. The bar is then removed, and the analyte is desorbed with a suitable solvent and analyzed. This technique offers high extraction efficiency even in large-volume samples, a large surface area for sorption, and minimal solvent usage. It also enhances selectivity when integrating MIPs as the adsorbent phase [108]. Cui et al. developed a molecularly imprinted nanofiber membrane-assisted SBSE technique for the selective analysis of sulfonamide antibiotics in animal feeds [109]. Selectivity studies of the nanofiber membrane were compared with aniline. In this work, the achieved LOD values were in the range 1.5 and 3.4 ng/g. The recovery values in different feed samples were found to be between 80 and 89%. The selectivity factor of the molecularly imprinted electrospun nanofiber membrane was found to be 2.7, indicating the selective binding ability of the nanofiber membrane.

### 3.2. Sensor-Based Detection

#### 3.2.1. Optical Sensors

Optical sensors are frequently used in pharmaceutical, biological, and environmental analyses due to their simplicity, low cost, and real-time monitoring capabilities [110,111,112]. Optical sensors detect changes in optical signals such as refractive index, absorbance, fluorescence, or Raman scattering resulting from the interaction between the analyte and the recognition element. Label-free optical detection enables analysis by directly measuring the physical or chemical changes that occur when the target molecule binds to the sensor surface. Since this method does not require sample pretreatment, it offers fast, practical, and real-time measurement. In contrast, labeled optical detection methods use a label such as a fluorophore, chromophore, or nanoparticle to detect the presence of the analyte. Labels provide signal formation and amplification and increase sensitivity, especially in the detection of low-concentration analytes. However, this labeled method usually requires a more complex preparation process and higher costs [111,113].

**Figure 4:**
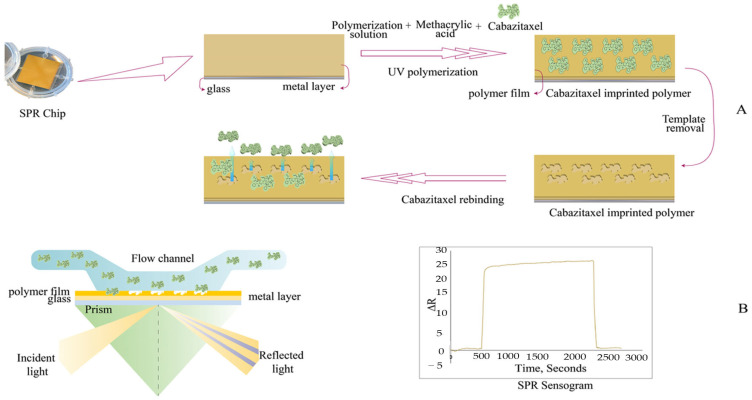
(**A**) Schematic representation of the preparation steps of the cabazitaxel-imprinted SPR sensor. (**B**) The measurement principle of the SPR-based sensor. (Reprinted with permission from [116]).

As an optical technique, surface plasmon resonance (SPR) measures the changes in refractive index on the metal surface of the sensor when the target molecule is bound or adsorbed onto the ligand-coated sensor surface. SPR sensors are widely used due to their simplicity, low cost, high sensitivity, and real-time measurement [114,115]. Recently, Aşır and colleagues developed a plasmonic-based selective sensor for determining cabazitaxel, a chemotherapeutic agent used in the treatment of metastatic prostate cancer [116]. Methacrylic acid (MAA), EGDMA, and AIBN were used as functional monomer, crosslinker, and initiator, respectively. The template monomer ratio was 1:3, and this optimum stoichiometric ratio was determined using a UV-vis spectrophotometer. The preparation steps of the cabazitaxel-imprinted SPR sensor and the measurement principle of the SPR-based sensor are given in Figure 4. The characterization results exhibited the successful functionalization of the sensor surface. The LOD value of the sensor was calculated to be 0.012 µg/mL, and it was found to have a wide linear detection range from 10 to 150 µg/mL (R^2^ = 0.9747). The fabricated SPR sensor exhibited outstanding selectivity in the presence of various competing drugs, including docetaxel, methylprednisolone, and dexamethasone. Additionally, recovery rates in artificial plasma were found to be between 97% and 99%. These values demonstrated the accuracy of the method and its applicability in biological samples. Cabazitaxel was also determined using HPLC. The SPR-based sensor does not require pretreatment or column conditioning, and therefore, it provides a more practical and easier analysis.

Tetracycline, which has broad-spectrum antimicrobial activity, is one of the most widely used antibiotics in both human and veterinary medicine due to its relatively low cost. It is also used as a growth promoter and feed additive in poultry and pigs. The determination of tetracycline in biological and environmental samples is important due to its widespread use and the risks associated with antimicrobial resistance and residue accumulation [117]. Bakhshpour-Yücel and colleagues developed a novel SPR sensor based on tetracycline-imprinted nanoparticles (Tc-MINps) for selective and real-time detection of tetracycline in milk samples [118]. The Tc-MINps was synthesized via microemulsion polymerization and immobilized on a bare gold SPR chip to form a recognition surface specific to tetracycline molecules. It was determined that the interaction between Tc-MINps and tetracycline molecules obeys the Langmuir-Freundlich isotherm model. The obtained detection limit was 0.45 mg/L. The sensor exhibited a high linear response (R^2^ = 0.979) in the concentration range of 0.5 to 20 mg/L in milk samples. The prepared sensor demonstrated consistent performance even when used for three consecutive days, and the relative standard deviation (RSD) value remained at a low level (1.44%). The developed sensor exhibited high specificity and selectivity against tetracycline even in the presence of similar antibiotics such as oxytetracycline, ciprofloxacin, and amoxicillin. The Tc-MINps-based SPR sensor provided label-free, low-cost, reusable, and real-time analysis for rapid and reliable determination of tetracycline in dairy products.

Therapeutic drug monitoring (TDM) is a critical clinical practice that enables individualized dose adjustment for effective and safe use of medication. It is vital to maintain the drug level in the blood between the minimum effective concentration and the minimum toxic concentration, especially for drugs with a narrow therapeutic range and a high risk of toxicity [119,120]. Fluorescence occurs when a substance absorbs light in the UV or visible range and then emits light at a lower energy as it returns to its ground state. Imatinib is a targeted drug used in the treatment of chronic myeloid leukemia and gastrointestinal stromal tumors and is classified as a tyrosine kinase inhibitor [121]. Hashem et al. developed a novel fluorescence sensor consisting of MIP-coated magnetite (Fe_3_O_4_) nanoparticles with a fluorescent probe (eosin) for the selective detection of imatinib [122]. When the MIP sensor was interacted with imatinib at different concentrations, the eosin molecules bound to the sensor surface were displaced by imatinib. The released eosin gave a fluorescent signal that depended on the imatinib concentration, and the imatinib calibration curve was obtained indirectly from this. They calculated the detection limit to be 0.93 µM with a linear range from 5 × 10^−6^ to 8 × 10^−4^ M. The sensor displayed a high reproducibility (RSD = <1.9%), repeatability (RSD = <2.1%), stability (over two months), and low cross-selectivity towards other ions and biomolecules. They also achieved high recovery rates for the determination of imatinib in pharmaceutical tablets and plasma.

Salbutamol is a bronchodilator in the β_2_-adrenergic agonist class, used especially in respiratory diseases such as asthma and chronic obstructive pulmonary disease. Salbutamol is used illegally as a feed additive to promote muscle growth, which poses a risk to consumer health. The World Anti-Doping Agency (WADA) has banned the use of salbutamol because it is used as a stimulant and anabolic agent [123]. Zhou et al. prepared a fluorescent sensor-based paper microfluidic chip system (FSU-BA@MIP) for the rapid and sensitive detection of salbutamol residues in food products [124]. Firstly, the filter paper was modified with SiO_2_ to create a porous structure. Then, a boronic acid-functionalized UiO-66-type MOF material was attached to the surface using the solvothermal method. Then, the polymer was obtained using the boronate affinity surface imprinting strategy, and salbutamol-specific recognition sites were formed as a result of washing the polymer with methanol. Salbutamol determination was performed with the prepared chips using a fluorescence-based analytical method. The prepared MIP layer specifically bound the salbutamol molecule. After this binding, a fluorescence signal was observed proportional to the amount of salbutamol with the reagents used (Ce^4+^ and Rhodamine B). A linear relationship was found between 0.001 and 5.0 mg/L. The LOD value of the method was calculated to be 0.019 µg/L. When different amounts of salbutamol were added to the pig samples, it was observed that the recovery values were within the acceptable range (63.22–85.83%). The developed method provided high selectivity and rapid results in the determination of salbutamol.

**Figure 5:**
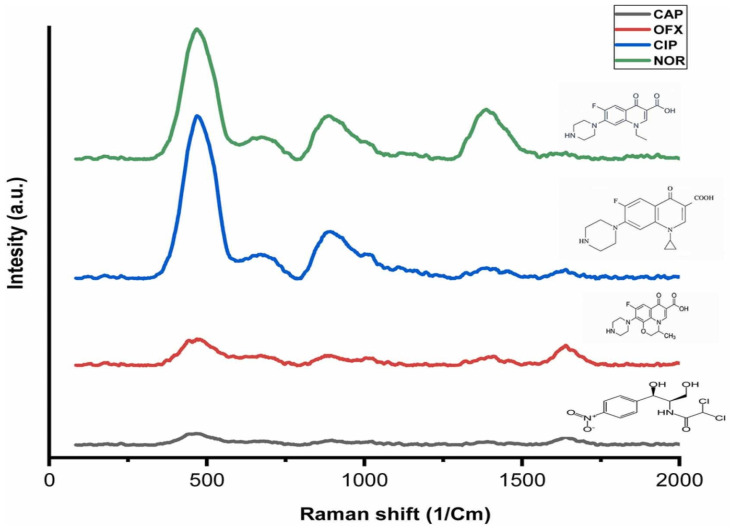
Comparison of the selectivity of norfloxacin imprinted SERS sensor (CIP: ciprofloxacin, OFX: ofloxacin, and CAP: chloramphenicol). (Reprinted with permission from [127]).

The Surface-enhanced Raman Scattering (SERS) method is one of the sensitive optical techniques that enable the detection of molecules at very low concentrations. Norfloxacin is a fluoroquinolone antibiotic that works by inhibiting DNA replication. Determining norfloxacin is important for food and environmental safety, as well as preventing antibiotic resistance [125,126]. Tarannum et al. developed a label-free, selective SERS-based MIP sensor to detect norfloxacin in milk [127]. The norfloxacin-imprinted polymer was created through radical polymerization using norfloxacin, MAA, EGDMA, and AIBN on the surface of a chitosan gold nanocomposite. The adsorption of norfloxacin onto the SERS sensor was examined at 1400 cm^−1^, and the peak intensity was found to correlate with the amount of norfloxacin adsorbed. The signal change was linear for norfloxacin concentrations ranging from 2.5 × 10^−4^ M to 2.5 × 10^−12^ M, based on the interaction with the SERS sensor. The detection limit was found as 2.5 × 10^−11^ M, which is below the limit values set by EU regulations. The selectivity of the SERS sensor against norfloxacin was compared with ciprofloxacin hydrochloride monohydrate, chloramphenicol, and ofloxacin. As shown in Figure 5, a norfloxacin-specific signal appeared at 1418 cm^−1^, but this signal disappeared after interaction with other antibiotics. This demonstrated that the norfloxacin-imprinted SERS sensor was selective only toward the target molecule.

The analytical performance of MIP-based optical sensors developed for the selective detection of various classes of drugs, including antibiotics (chloramphenicol, ofloxacin, amoxicillin, benzylpenicillin), a natural antioxidant (quercetin), an anticancer drug (etoposide), and illicit drugs (methamphetamine and cocaine), is presented in Table 2.

#### 3.2.2. Electrochemical Sensors

In MIP-based electrochemical sensors, the MIP layer immobilized on the surface of the transducer functions as a recognition element. The interaction between the target molecule and the imprinted sites within the MIP layer produces a measurable change in the electrical signal of the transducer. MIP-based electrochemical sensors have better sensitivity, selectivity, stability, robustness, and longer shelf life compared to conventional biosensors. In recent years, MIP NPs have gained an important place in electrochemical sensing due to their high selectivity and recognition ability for target molecules. The integration of MIP NPs with various electrode types has enabled the development of highly sensitive and selective electrochemical platforms for pharmaceutical [138,139,140], environmental [141,142], and clinical applications [10,143,144]. Carbon paste electrodes (CPEs), glassy carbon electrodes (GCEs), pencil graphite electrodes (PGEs), and screen-printed electrodes (SPEds) are among the commonly used electrode platforms. These electrode types are usually modified with conductive polymers (e.g., polypyrrole (Ppy), polyaniline (PANI), polythiophene (PT), poly(3,4-ethylenedioxythiophene) (PEDOT)) [145], and nanomaterials (e.g., multi-walled CNTs, graphene oxide (GO), carbon dots (CDs), or metal nanoparticles) to increase the electron transfer rates, surface area, and chemical stability of the sensing interface [11,69,146]. MIP NPs-based electrochemical sensors provide excellent performance in terms of sensitivity, response time, and stability in complex sample matrices. These features make them prominent for the selective determination of pharmaceutical compounds and drugs of abuse in complex biological and environmental samples [147].

Among the types of electrochemical sensors, potentiometric sensors measure the potential difference based on the activity (concentration) of ions or molecules in a solution using ion-selective electrodes (ISEs). The indicator electrode contains a membrane that is selective for a particular type of ion or molecule. The measured potential has a logarithmic relationship with the concentration of the ion or molecule, as described by the Nernst equation. It is often preferred in environmental, biological, pharmaceutical, and food analysis due to its advantages, such as low energy requirement, structural simplicity, long lifespan, and high selectivity [148]. MIP NPs are widely used as recognition elements in potentiometric analysis [149].

Clenbuterol is a veterinary drug that makes breathing easier by widening the airways, but it is used illegally by athletes because it helps burn fat faster by speeding up the metabolism [150]. Özkütük et al. prepared the MWCNT@MIP-based CPE and poly(vinyl chloride) (PVC) membrane electrodes as indicator electrodes for the selective potentiometric detection of clenbuterol [151]. They obtained polymers via thermal polymerization in the presence of MWCNT, clenbuterol, diethylenetriaminepentaacetic acid-modified chitosan-Dy(III), and epichlorohydrin. Then, they removed clenbuterol from the polymer using methanol/acetic acid solution and obtained metal-chelate-based MIP NPs. They also synthesized the NIP under the same polymerization conditions, without the target molecule, to evaluate the binding efficiency of the imprinted polymer to the target molecule. They prepared two different sensors, one containing PVC and the other containing CPE, for the MIP. Clenbuterol determination displayed a wider linear range (10^−4^–10^−9^ mM) with the MWCNT@MIP@CPE sensor compared to the MWCNT@MIP@PVC sensor. The NIP sensors gave lower and more stable potential values in clenbuterol solutions than MIP sensors. Since NIP sensors lack specific recognition sites for clenbuterol, interactions with different concentrations of clenbuterol resulted in significantly lower potential changes. The stable potential response was obtained at 1 and 2 min with MWCNT@MIP@CPE and MWCNT@MIP@PVC electrodes, respectively. They reported that no significant potential loss was observed when the sensors were reused ten times.

**Figure 6:**
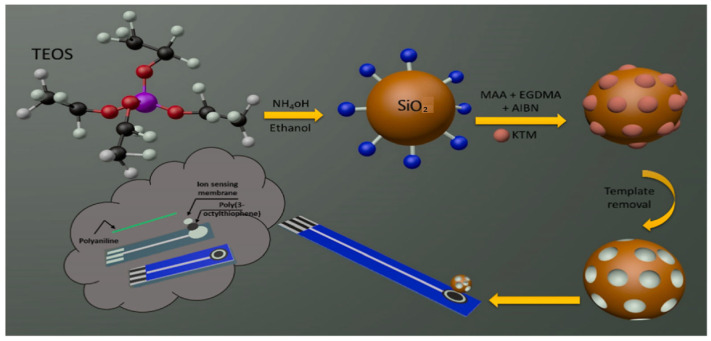
Schematic representation of the imprinting process and sensor membrane. (Reprinted with permission from [152]).

Soliman et al. fabricated a potentiometric sensor for the selective determination of ketamine in plasma, urine, and beverages [152]. Ketamine, an effective anesthetic and analgesic drug, is misused due to its psychoactive effects. To prepare the working electrode, the carbon screen-printed electrode (C-SPEd) was modified using CV with PANI as the conductive polymer layer. The MIP layer in the shell structure was formed around silica nanoparticles and incorporated into the PVC membrane as an ionophore (Figure 6). The prepared sensor (C-SPEd/PANI/MIP) responded linearly in the concentration range from 10^−6^ M to 10^−2^ M and had a stable potential over a wide pH operating range (pH 2–5). PANI, a conductive polymer, facilitates electron transfer and provides electrochemical stability. The response time of this sensor was quite fast (3–5 s). The selectivity of the sensor was evaluated using the separate solution method and showed good selectivity in the presence of quetiapine and common ionic species (Na^+^, K^+^, Ca^2+^, Mg^2+^, and Zn^2+^).

Differential Pulse Voltammetry (DPV) is a sensitive electrochemical technique used in the determination of electroactive substances. In this method, a linearly increasing potential is applied to the working electrode, while a short-term pulse is given at each potential step. The current differences measured before and after each pulse are recorded, and a current-potential graph is obtained. Since the noise is low, analytes can be detected even at very low concentrations. Çetinkaya et al. prepared an electrochemical sensor (ABR/4-ABA/ZnNFs@GO/MIP/GCE) for the selective determination of abrocitinib, which is used for the treatment of atopic dermatitis [153]. They synthesized zinc nanoflowers (Zn NFs) from plant extract using a green method and then conjugated them with GO. They carried out the polymerization around the ZnNFs@GO core using 4-aminobenzoic acid (4-ABA), EGDMA, and 2-hydroxy-2-methylpropiophenone as functional monomer, crosslinker, and photoinitiator, respectively. They performed the determination of abrocitinitib by indirect measurement using the [Fe(CN)_6_]^3−/4−^ as a redox probe with DPV. The developed sensor exhibited a wide linear detection range (1.0 × 10^−13^ to 1.0 × 10^−12^ M) and an impressively low detection limit (2.18 × 10^−14^ M). The sensor showed high selectivity towards abrocitinib in the presence of similar (ibrutinib, ruxolitinib, tofacitinib, zonisamide, and acetazolamide) and interfering compounds (ions, dopamine, uric acid, ascorbic acid, and paracetamol), confirming the effectiveness of molecular imprinting.

In a study published by Kutner, an electrochemical MIP NPs-based chemosensor was successfully prepared for the selective determination of cilostazol, which is used for the treatment of peripheral arterial disease [154]. The MIP NPs were obtained using MAA, EGDMA, and 2,2′-Azobis(2,4-dimethylvaleronitrile (ABDV) as functional monomer, crosslinker, and initiator, respectively. The MIP NPs-based chemosensor demonstrated broad linearity in the concentration range between 134 nm and 2.58 μM. As a result of DPV and electrochemical impedance spectroscopy (EIS) analysis using ferrocene as a redox probe, the detection limits were found as 93.5 nM and 86.5 nM, respectively.

**Figure 7:**
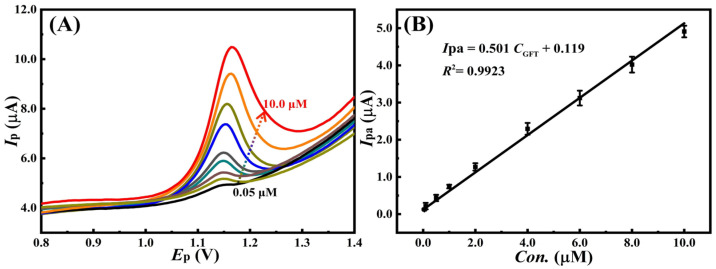
(**A**) DPV responses at different concentrations of Gefitinib, (**B**) Calibration curve for Gefitinib detection (Reprinted with permission from [155]).

Huang et al. developed the MIPs@CNTs sensor in a single step using a hydrothermal method for the detection of gefitinib with the DPV [155]. Gefitinib is a tyrosine kinase inhibitor used in the treatment of lung cancer. The synthesized sensor material was characterized using scanning electron microscopy (SEM), atomic force microscopy (AFM), Fourier transform infrared (FT-IR), CV, and EIS. They calculated the detection limit value for gefitinib as 0.014 µM. They reported that the prepared sensor was highly sensitive and selective in a wide concentration range (0.05–10.0 µM) (Figure 7). They also demonstrated the analytical performance of the sensor in the pharmaceutical formulations and rat plasma samples for the selective detection of gefitinib.

Square wave voltammetry (SWV) is an electrochemical method that measures the current resulting from oxidation and reduction reactions on the electrode surface. In this technique, the applied potential is changed rapidly and continuously in the form of a square wave. The concentration of the analyzed substance is determined by measuring the current. This method is quite fast and is as sensitive as DPV. Afzali et al. developed an MIP-coated electrochemical sensor containing Fe_3_O_4_ and GO to selectively determine capecitabine [156]. Capecitabine is a fluoropyrimidine carbamate used in various types of cancer, including colorectal, breast, and pancreatic cancer. Like other chemotherapy drugs, it can cause serious side effects [157]. The GCE surface was modified with Fe_3_O_4_@GO@MIP [156]. As the capecitabine concentration increased, a linear increase in the current signals was observed. They reported that the capecitabine-imprinted electrochemical sensor exhibited linearity from 1.0 to 100.0 nM with a detection limit of 0.324 nM. The RSD value of independent sensors prepared at different times was found to be 1.25%. This value indicated that the sensor maintained consistent performance across different production stages. The prepared sensor maintained 98.5% of the initial response of the capecitabine reduction peak current after 30 days, demonstrating the long-term stability of the prepared sensor.

Garcia and coworkers prepared a new electrochemical sensor with high selectivity for the determination of folic acid using the SWV [158]. Magnetic nanoparticles (Fe_3_O_4_) synthesized by the polyol method were used as cores and then coated with MIP. Folic acid was removed from the polymer by Soxhlet extraction using a mixture of methanol and NaOH solution. After mixing GO, Fe_3_O_4_@MIP, and graphite powder, mineral oil was added, and the resulting paste was placed on the tip of the electrode to prepare the sensor. The prepared sensor was characterized using SEM, FT-IR, Brunauer-Emmett-Teller (BET), and X-ray diffraction (XRD). The maximum adsorption capacity for folic acid imprinted magnetic polymer and the non-imprinted polymer was found to be 30.4 mg/g and 15.3 mg/g, respectively. The LOD value of the sensor was calculated as 0.65 µM, and the linear range was 2.5–48 µM. The developed sensor was successfully applied to drug samples and river water, and recovery rates were found between 96% and 100%. When compared with the HPLC-UV method, the results are almost exactly consistent. The repeatability of the developed sensor was found to be only 2.1% RSD in 10 consecutive measurements under the same conditions. This shows that the method gives reliable and stable results.

The analytical performance of MIP-based electrochemical sensors developed for the detecting of various classes of drugs, including cardiovascular system drugs (losartan, and amiodarone), an antidepressant drug (fluoxetine), antipsychotic drugs (lurasidone hydrochloride, chlorpromazine), antiviral drugs (favipiravir, tenofovir, daclatasvir, umifenovir), cancer drugs (ruxolitinib, dasatinib, bortezomib), urinary system drugs (alfuzosin and solifenacin), antibiotics (erythromycin, cefdinir, norfloxacin, ofloxacin), and illicit drugs (3,4-methylenedioxypyrovalerone, and cocaine), are presented in Table 3.

### 3.3. Controlled Drug Release

Research on drug development is not limited to the discovery of new molecules. It also focuses on improving the drug delivery system. A drug delivery system (DDS) enables the transport and release of therapeutic agents within the body. The main purpose of this system is to maintain the drug level in the blood between the lowest effective concentration and the lowest toxic concentration. Traditional DDSs often face problems such as poor drug absorption, rapid delivery, low bioavailability, and short therapeutic effect [178]. Hydrogels, micro- or nanoemulsions, nanocarriers, liposomes [179], micelles [180], dendrimers, microcapsules [181], and polymer carriers [182] can be used as DDS. Traditional polymers can cause rapid drug release at high doses. Their stability in DDS is not sufficient. MIP NPs become prominent for their unique ability to recognize target molecules with high selectivity compared to other nanocarriers. Solid lipid nanoparticles are non-toxic and relatively more stable as they can be naturally degraded compared to liposomes and polymeric nanoparticles [183,184]. Liposomes and polymeric nanoparticles are biocompatible and approved nanocarriers for clinical use. Copaxone^®^, Neulasta^®^, and Eligard^®^ are polymeric nanoparticles approved by the U.S. Food and Drug Administration (FDA). Myocet^®^, Daunoxome^®^, Doxil^®^/Caelyx^®^, and Onivyde^®^ are FDA-approved liposomal nanocarriers. However, liposomes have disadvantages such as low encapsulation capacity of lipophilic drugs, frequent leakage, and instability in biological fluids and aqueous solutions [185]. MIP NPs lag behind lipid and polymer-based systems in terms of biodegradability, large-scale production, and regulatory approval. Drug carriers are required to function in an aqueous environment to exhibit effective release behavior. Therefore, the preparation of water-compatible MIP NPs offers advantages in DDS.

A controlled drug delivery system (CDDS) provides an effective treatment method by providing targeted delivery of drugs in the desired dose range. These systems regulate the speed and timing of drug delivery to the body, thus optimizing treatment results and minimizing the risk of side effects. Therefore, CDDS is considered an important component of modern drug treatments. In recent years, significant interest has been shown in the development of CDDS that aim to minimize drug dosage, extend dosing intervals, and increase the efficacy and comfort of patient treatment by reducing side effects [186].

MIPs have gained attention in CDDS due to their high selectivity toward the target molecule. The selectivity of MIPs towards the target molecule increases the efficiency of drug loading into the polymer. Compared to natural drug carriers, MIPs offer excellent chemical, thermal, and mechanical stability, making them suitable for harsh biological conditions [14,24].

The synthesis of MIPs can be achieved relatively easily and cost-effectively using well-known polymerization methods such as precipitation, bulk, suspension, or surface imprinting techniques. Molecular imprinting-based CDDS can be easily synthesized as nanoparticles [187], microspheres [188], films, gels [15], nanofibers [189], and nanospheres [190]. Therefore, it can be adapted for different administration routes, such as oral, intravenous [191], ocular [192,193], and transdermal [194]. The nanoscale nature of the developed carrier system provides a significant advantage, particularly in targeting cancer therapy. The small size of the nanoparticles enhances their accumulation in tumor tissue by leveraging the enhanced permeability and retention (EPR) effect, thus facilitating intercellular transport and supporting intracellular uptake. Drug release from MIP-based carriers depends on factors like the type of reagents (such as cross-linkers, monomers, drug compounds, initiators, and solvents), polymer composition, and synthesis method. These parameters should be optimized to ensure effective and controlled drug release.

Target molecules can be removed from MIPs in a controlled manner by various exogenous and endogenous stimuli [195] such as pH, temperature, magnetic field, light [196], redox conditions [18], peptides, proteins, and enzymes. This property is beneficial in tumor-targeting therapies where acidic pH or external magnetic fields are used to trigger drug release. Thus, drugs bound within the MIP cavities are usually released in a controlled and time-dependent manner. This allows for prolonged therapeutic effects and reduced dosing frequency [197]. Han and colleagues developed a dual-responsive UiO-66-MIP-based drug delivery system that enables selective targeting of tumor cells and controlled release of doxorubicin in response to acidic pH and glutathione levels in the tumor microenvironment [198]. In the study of Kubo et al., a dual-responsive MIP based on a magnetic-thermal core coated with a thermally responsive polymer layer was prepared for the release of methotrexate. This prepared polymer showed significantly enhanced drug release when exposed to both thermal and alternating magnetic fields [199]. Yari-Ilkhchi and co-workers prepared a pH-responsive MIP-based nanocarrier system for the targeted delivery of sodium thiopental, and its release behavior was evaluated in simulated gastric fluid and simulated intestinal fluid, achieving controlled drug release under intestinal pH conditions [200].

Drug release kinetics are mathematical models used to study the effects and release of drugs in in vivo and in vitro environments. The zero-order model describes how the amount of a drug changes over time after it is given to the body. It assumes that the drug is released at a constant rate [201]. According to the first-order model, the reaction rate or drug release rate varies depending on the concentration of the reactant or drug. In this model, the amount of drug decreases exponentially with time [178]. The Higuchi Model, developed by Higuchi in 1961, is frequently used in drug release kinetics [202,203]. It is based on the dissolution of the drug in the matrix and the diffusion process within the matrix. This diffusion process causes the release of the drug from the matrix in increasing amounts proportional to the square root of time. The Korsmeyer-Peppas mathematical model is a comprehensive model that describes the processes of water diffusion into the matrix, matrix swelling, and polymer matrix dissolution. This model is useful in situations where the mechanism of drug release is unclear or where multiple release mechanisms are involved simultaneously [15,178,204].

MIP-based DDS systems are characterized by their advantages of high selectivity, high drug loading, and controlled drug release. However, they should be prepared using biocompatible, non-toxic, and biodegradable materials for use in drug delivery applications. Various methacrylate-based cross-linkers and functional monomers such as EGDMA, MBA, 2-hydroxyethyl methacrylate (HEMA), and MAA have been widely utilized in designing MIP-based drug delivery platforms due to their favorable biocompatibility and acceptable toxicity profiles [24,205]. However, no studies have been conducted to investigate the long-term toxicity and metabolic pathways of these substances. The long-term toxicity of MIP NPs has not been fully elucidated [24,200]. Nonetheless, their long-term metabolic behavior and potential toxicological impacts still require comprehensive investigation.

Various studies have focused on the use of substances such as β-cyclodextrin [206], chitosan [207], dopamine [187], glucose, and methacrylated fructose [191] as functional monomers and crosslinking agents to enhance biocompatibility. Liu et al. developed a TiO_2_-doped chitosan oligosaccharide-based polymer for the controlled release of salidroside. This biocompatible system was tested for cytotoxicity using IPEC-J2 cells [207]. They found a cell survival rate of 120 ± 12.46% when MIP NPs were used at a concentration of 1000 μg/mL. This indicates that the prepared MIP NPs had no cytotoxic effects. The increase in cell viability can be attributed to TiO_2_, as cell viability also increased when cells were treated with various concentrations of TiO_2_ alone. However, the long-term effects of the synthesized MIP NPs on cell viability should be observed.

In a reported study published by Ali et al., glucose was used as a natural crosslinker in the synthesis of docetaxel-loaded magnetic MIP, thus providing safer and more effective drug delivery by imparting biodegradability and biocompatibility to the nanocarrier [87]. Magnetic MIPs were treated with acidic and basic solutions for 10 days, and their degradation was examined in vitro. Greater degradation was observed for magnetic MIPs in the acidic environment, while degradation was also detected in the basic environment. In the study by Ezati et al., serotonin-decorated MIP NPs were synthesized for irinotecan delivery [208]. In this study, the degradation behavior of MIP NPs was investigated in vitro over a 12-day period. When the degradation of MIP NPs at pH 3, 7.4, and 11 was examined, the best degradation was obtained at pH 3. In both studies, the biodegradability of the crosslinker facilitated the degradation of the synthesized MIP NPs. Reducing the amount of crosslinker resulted in faster degradation of the polymer. However, these results may deviate significantly under complex biological conditions in the body. Additionally, the experimental duration was insufficient; extending the observation time is necessary to obtain a more comprehensive understanding of the degradation process of MIP NPs.

Polyethylene glycol, lactic acid, and polylactic glycolic acid (PLGA) are biodegradable. Biodegradable polymers are broken down by hydrolysis into non-toxic metabolites. The use of biodegradable reagents that enable polymer degradation is very promising for MIP NPs. For example, Gagliardi and co-workers were able to prepare completely degradable PLGA-based MIP NPs [209]. Most studies have examined only cell viability in vitro. In these studies, MIPs generally showed no in vitro toxicity when biodegradable reagents were used. Further studies of their in vivo applications are necessary to better evaluate biodegradability and toxicity [210,211]. Kassem et al. observed in their studies that MIP NPs suppressed for EGFR could remain in tissues without any significant toxic effects [210]. In order to obtain reliable results, long-term evaluation through in vivo studies, including toxicity testing, is necessary. In addition to toxicity testing, inflammatory response and changes in cell morphology should also be observed. In vivo studies on MIPs should also consider the formation of a protein corona, as this phenomenon can alter their bioavailability and therapeutic performance [212].

The use of biodegradable crosslinkers can facilitate polymer degradation, reducing the accumulation of MIP NPs in the body. The amount of crosslinker affects both the drug release from MIP NPs and the accumulation of these nanoparticles in the body [28]. Reducing the amount of crosslinkers allows the MIP NPs to degrade more easily. Thus, the accumulation of the MIP NPs in the body is reduced, and cytotoxicity can be reduced. However, using a small amount of crosslinker will also cause the MIP NPs to have a looser structure, resulting in a short-term release of the drug. This is undesirable for CDDS. The use of high amounts of crosslinkers results in a harder polymer structure that cannot be broken down in the body over an extended period, potentially increasing toxicity. Therefore, the amount of crosslinker and its biodegradability are important parameters.

The particle size of nanoparticles affects drug loading, release, and toxicity. Small nanoparticles are beneficial for drug release kinetics, but tend to accumulate in the biological medium. Particles larger than 200 nm may cause capillary occlusion. Particles in the 10–150 nm range provide effective tissue/cell penetration and longer circulation times [213].

The surface charge of MIP NPs also plays a role in toxicity. Positively charged MIP NPs can cause hemolysis and coagulation. Anionic and neutrally charged nanoparticles are preferred in these systems [211]. For effective drug release in the body, drug carriers generally need to function in aqueous environments [24].

MIP nanocarriers prepared for therapeutic purposes exhibit efficient drug loading, transport, and release profiles. Cytotoxicity, biocompatibility, and biodegradability affect their safety profile. To prevent the long-term toxicity of MIP NPs, the use of biodegradable reagents offers a solution, as they will effectively degrade the polymer. It should be reiterated that further research is necessary to assess their long-term safety, toxicity, and biocompatibility. In these studies, it is necessary to improve biocompatibility, reduce cytotoxic effects on healthy cells, increase biodegradability, and ensure long-term safety. Some studies have detailed the in vitro cell toxicity of MIP-based DDSs. However, in vivo evaluations are necessary to determine their clinical safety. Therefore, further studies on the development and evaluation processes of MIP-based DDSs are needed to evaluate their safety profiles and comply with biomedical regulations.

A summary of some MIP NPs-based drug delivery systems is given in Table 4.

#### 3.3.1. Drug Release Systems for Cancer

The use of MIPs for the controlled and targeted release of cancer drugs has been intensively investigated in recent years [195,217,218]. Since MIPs can be designed to recognize a specific cancer drug (e.g., methotrexate, doxorubicin), the carrier can be ensured to interact strongly and selectively only with the relevant molecule. The microenvironment of cancer cells is typically acidic, and the amount of glutathione is high. Designing MIPs sensitive to these conditions can ensure drug release only in the tumor environment. Thus, specific targeting of cancer cells can be achieved, reducing damage to healthy tissue.

**Figure 8:**
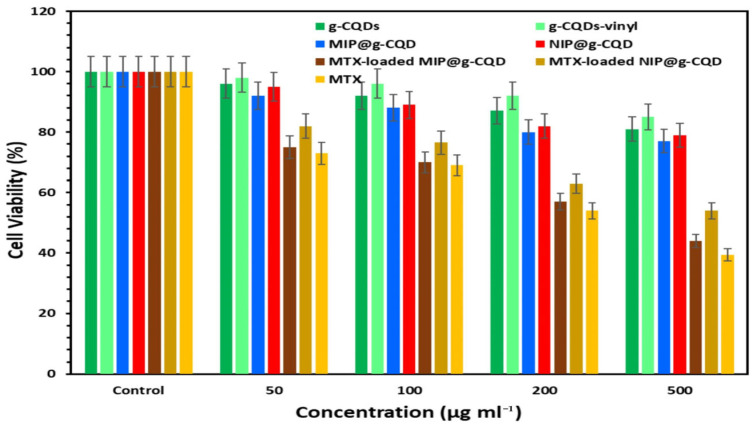
In vitro cell viability of MCF-7 breast cancer cells after 48 h incubation with various concentrations of pure g-CQD, vinyl modified g-CQD, methotrexate, non-methotrexate-loaded and methotrexate-loaded NIP@g-CQD, non-methotrexate-loaded and methotrexate-loaded MIP@g-CQD (Reprinted with permission from [219]).

Chemotherapeutic agents such as doxorubicin and methotrexate have narrow therapeutic effects as well as high toxicity. The development of biocompatible drug delivery systems is of great importance in reducing the side effects and increasing the therapeutic efficacy of toxic drugs commonly used in cancer and autoimmune diseases [181]. Nasiriani et al. proposed a pH-sensitive and photoluminescent nanocarrier system for methotrexate [219]. A polymer was synthesized via precipitation polymerization using vinyl-functionalized gelatin-based carbon quantum dots (g-CQDs) as the core, with acrylamide as the functional monomer, 1,4-bis(acryloyl)piperazine as the cross-linker, APS as the initiator, and methotrexate (MTX) as the template. The loading capacity of the MIP@g-CQD (80.14%) was significantly higher than that of the NIP@g-CQD (41.2%). Approximately 75% of methotrexate was released from MIP@g-CQD after 280 h at pH 5.0. However, significantly lower release was observed at pH 7.4, indicating minimal exposure to healthy tissues. The release mechanism followed Fickian diffusion. As shown in Figure 8, the MTT assay on MCF-7 cells revealed that after 48 h of incubation, MTX-loaded MIP@g-CQD reduced cell viability to approximately 40%, indicating effective cytotoxicity against breast cancer cells. In contrast, drug-free MIP@g-CQD maintained over 80% cell viability. Additionally, MTX-loaded MIP@g-CQD was less toxic than free MTX, suggesting that controlled release reduces off-target effects. Drug-free MIP@g-CQD has not been studied in healthy cells, and other toxicity tests have not been conducted. A better understanding would be obtained if MTX-free MMIP@g-CQD nanoparticles were tested in healthy cells.

Liu and colleagues prepared targetable and biocompatible MIP NPs using the epitope of the epidermal growth factor receptor (EGFR) as a template, combining chemotherapy and photothermal therapy [19]. They obtained highly biocompatible MIP NPs (DOX@MIP) by surface polymerization of dopamine around doxorubicin-loaded ZIF-8 particles in a weakly basic environment. They used the EGFR epitope to recognize and target cancer cells with high specificity. While a 10% doxorubicin release performance was obtained from DOX-MIP in 100 h at pH 7.4, 6 times more release was obtained in 29.5 h at pH 5. In addition to pH-dependent release, DOX@MIP NPs also exhibited excellent photothermal conversion under NIR laser light, resulting in increased doxorubicin release. Furthermore, DOX@MIP NPs did not show structural degradation even after 5 irradiation/cooling cycles. When high concentrations of MIP and NIP nanoparticles were applied to 16HBE cells, a human bronchial epithelial cell line, cell viability remained above 90%. This result demonstrated the high biocompatibility of MIP and NIP. DOX-loaded MIP NPs showed significant cytotoxic effects on A549 cells, and this cytotoxic effect increased in NIR light and acidic medium. This targeted, pH and NIR stimulated MIP-based nanocarrier system is a promising platform for effective and safe cancer treatments.

#### 3.3.2. Drug Release Systems for Diabetes

**Figure 9:**
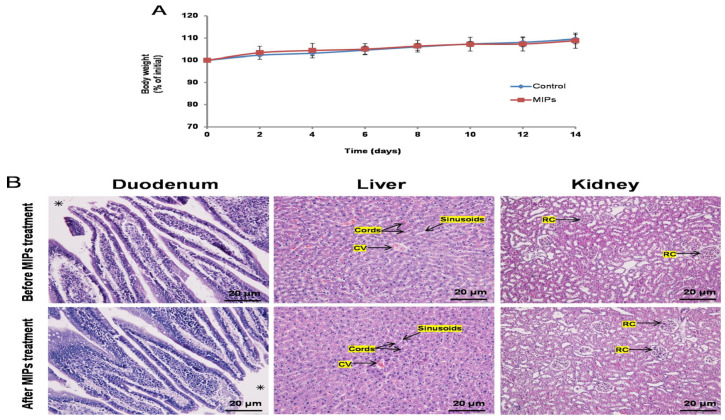
(**A**) Changes in body weight of mice in the control and MIP-treated groups over time, and (**B**) representative hematoxylin and eosin-stained histological sections of intestine, liver, and kidney before and after treatment with MIP NPs. CV: central vein; RC: renal corpuscle (glomerulus and granular capsule). Asterisks indicate the duodenum lumen. (Reprinted with permission from [220]).

Insulin is a difficult molecule to administer orally due to its low stability and poor absorption in the gastrointestinal tract. Many diabetic patients encounter problems such as dosage measurement errors and pain at the injection site when using insulin pens. These make it important in the development of drug delivery systems for oral administration. Paul and colleagues prepared insulin-imprinted nanoparticles to enable effective oral delivery of insulin from the intestines to the blood [220]. They used MBA as a crosslinker and MAA and N-hydroxyethyl acrylamide as functional monomers. They examined the binding of insulin to MIP NPs using fluorescence spectroscopy and found that MIP NPs showed high selectivity towards insulin. The loading capacity of MIP NPs was 55.75 mg/g, and the loading efficiency was calculated as 82.13%. Insulin release was monitored for 24 h in PBS at pH 7.4. Under these conditions, the release percentage of insulin from MIP NPs was determined to be 45% in the presence of tissue lysate and 25% without it. In contrast, the amount of insulin released from NIP nanoparticles was approximately 2.4 times higher than that from MIP NPs. In vivo absorption of insulin was evaluated in streptozotocin-induced diabetic rats, and it was shown that insulin-loaded MIP nanoparticles effectively reached systemic circulation by crossing the intestinal epithelium via the transcellular route. As seen in Figure 9A, diabetic rats treated with insulin-loaded MIP for 14 days showed an increase in body weight similar to the control group. In Figure 9B, no damage or toxic effects were observed in the intestine, liver, and kidney tissues. These findings demonstrated that insulin-imprinted MIP NPs can be administered orally.

#### 3.3.3. Drug Release Systems for Neurological Diseases

Donepezil is a centrally active, rapid, and reversible acetylcholinesterase inhibitor approved by the US Food and Drug Administration (FDA) for the treatment of Alzheimer’s disease [221,222]. In a study by Figueiredo and colleagues, donepezil-imprinted nanoparticles were prepared that can cross the blood-brain barrier and deliver donepezil to the brain for a longer period than existing nanocarriers [223]. MIP NPs were synthesized via radical polymerization using donepezil, methacrylic acid, AIBN, and EGDMA. They were then functionalized with apolipoprotein E based on avidin-biotin interaction and labeled with fluorescent biotin. The maximum adsorption capacities of donepezil on MIP and apolipoprotein E-functionalized MIP NPs were 142 mg/g and 105 mg/g, respectively. It was found that the binding of donepezil to the MIPs followed the Sips adsorption isotherm, which indicates the heterogeneous nature of the surface. All nanoparticle groups exhibited hemolytic activity below 15%, indicating they did not damage blood cells. In MTT and lactate dehydrogenase assays conducted with both brain capillary endothelial cells (hCMEC/D3) and fibroblast cells (L929), cell viability remained above 70%, demonstrating that the nanoparticles possessed low cytotoxicity. In vivo studies showed that MIP and MIP-apolipoprotein E nanoparticles could sustain the release of donepezil in plasma for at least 8 h, indicating potential for improved therapeutic efficacy. The authors concluded that MIP and MIP-apolipoprotein E nanoparticles can be considered as good candidates for the controlled and sustained delivery of donepezil in Alzheimer’s patients.

In recent years, dopamine has been frequently used as a functional monomer in polymerization processes due to its biocompatibility and the presence of functional groups such as catechol and amine. It interacts non-covalently with template molecules through hydrogen bonds, electrostatic forces, and π-π interactions [224]. In another study by Khaneghah and colleagues, magnetic MIP NPs were synthesized for the controlled release of rivastigmine, used to treat Alzheimer’s and Parkinson’s diseases [225]. Dopamine was self-polymerized in a basic environment to form a polymer coating on the graphene oxide/Fe_3_O_4_ nanocomposite. The maximum loading capacity of the prepared magnetic MIP NPs was obtained to be 39.24 mg/g. The drug loading was found to fit the Freundlich isotherm. The highest drug release percentage was found to be 78.65% at pH 9 for 120 min. From the drug release kinetic analysis, it was observed that MIP NPs exhibited Fickian diffusion behavior. This indicates that the drug is released directly proportional to the concentration.

Trotta and colleagues developed molecularly imprinted cyclodextrin nanosponges (MIP-NSs) that can provide controlled release of (S)-2-amino-3-(3,4-dihydroxyphenyl) propanoic acid (L-DOPA), a drug used in the treatment of Parkinson’s disease [226]. They found that MIP-NSs released L-DOPA biphasically, with approximately 32% of the drug released within the first 6 h and a cumulative release of 52.6% within 48 h. Pseudo-zero-order kinetics were observed during the first 4 h. After 4 h, they achieved a slower and more controlled release. These results suggest that MIP-NSs provide a promising approach to achieve sustained and controlled delivery of L-DOPA, which may help improve the treatment of Parkinson’s disease.

#### 3.3.4. Drug Release Systems for Infection

MIP-based drug delivery systems are among the smart systems being researched increasingly and developed for treating infectious diseases. These systems enable targeted and controlled drug release, aiming to enhance treatment effectiveness and provide a strategic approach to global health challenges like antibiotic resistance. Ma et al. successfully achieved controlled tetracycline release in response to environmental temperature changes using an MIP NP with thermal and magnetic sensitivity [227]. Prusty and co-workers developed a biocompatible, temperature-responsive hybrid hydrogel system around ZnO nanoparticles for the controlled release of ciprofloxacin, which exhibits high antibacterial activity [228].

**Figure 10:**
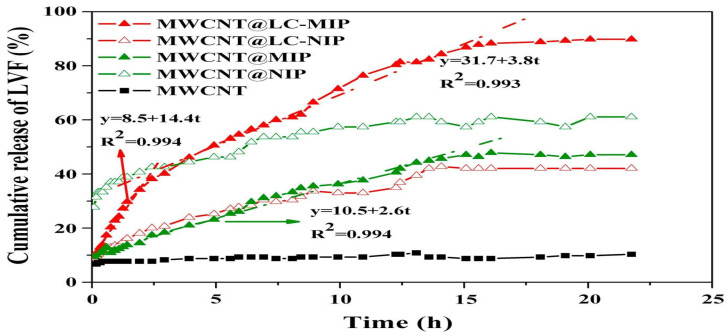
Comparison of release profiles of Levofloxacin from MWCNT@LC-MIP, MWCNT@LC-NIP, MWCNT@MIP, and MWCNT@NIP at 100 µg/mL in acetonitrile dissolution. (Reprinted with permission from [229]).

Levofloxacin is a third-generation fluoroquinolone antibiotic commonly used to treat infections of the lungs, urinary tract, skin, and soft tissues, thanks to its broad antibacterial spectrum. Since levofloxacin is rapidly absorbed after oral administration with a short biological half-life, the development of controlled-release systems is clinically important. In the study of Zhang et al., a molecularly imprinted drug carrier (MWCNT@LC-MIP) was successfully developed using a liquid crystalline monomer for the controlled release of levofloxacin [229]. Polymerization was carried out around the multi-walled carbon nanotube functionalized with 9-vinylanthracene, using AIBN as an initiator, EGDMA as a crosslinker, and MAA together with 4-Methylphenyl dicyclohexyl ethylene (MPDE) as functional monomers. The highest drug loading was achieved with a 300 µg/mL solution, resulting in a loading capacity of 26.0 mg/g and an entrapment efficiency of 86.8%. They observed zero-order release for levofloxacin imprinted drug carriers both with (MWCNT@LC-MIP) and without (MWCNT@MIP) the use of liquid crystal as monomer. Biphasic drug release was achieved from MWCNT@LC-MIP loaded with levofloxacin (Figure 10). During the first 1.92 h, levofloxacin was desorbed due to nonspecific binding, followed by sustained release through Fickian diffusion, which lasted up to 21.75 h. In contrast, desorption of levofloxacin from MWCNT@MIP occurred in a single phase, with the release completed in 15 h. The in vivo pharmacokinetic study showed that the relative bioavailability of MWCNT@LC-MIP, MWCNT@MIP, and MWCNT was 578.9%, 58.0% and 11.7%, respectively. In conclusion, the use of MWCNT@LC-MIP enhanced the bioavailability and sustained release properties of levofloxacin for oral administration.

Ritonavir is an antiretroviral protease inhibitor widely used in the treatment of human immunodeficiency virus (HIV) infection. Controlled release of ritonavir provides a major clinical advantage in HIV treatment by increasing treatment efficiency, reducing side effects, and reducing dosing frequency. Arıkan et al. prepared ritonavir imprinted poly-2-hydroxyethyl methacrylate N-methacryloyl-L-tryptophan methyl ester [p(HEMA-MATrp)] nanoparticles for nasal release of ritonavir [230]. The p(HEMA-MATrp) MIP NPs were synthesized via mini-emulsion polymerization in the presence of EGDMA (crosslinker), APS (initiator), and HEMA and MATrp as monomers. From in vitro drug release studies, cumulative release rates were found to be 26% at pH 4.0 and 32% at pH 7.4. Kinetic analysis showed that the release profiles best fit the Korsmeyer-Peppas model, indicating a controlled and multidirectional Fickian diffusion mechanism across all concentrations. Cytotoxicity evaluation via MTT assay demonstrated that the synthesized MIP NPs exhibited low cytotoxicity and excellent biocompatibility in the L929 mouse fibroblast cell line. When cell viabilities were examined after 24 and 48 h, it was observed that as the concentration of ritonavir-imprinted NPs was increased, the percentage viability decreased most significantly in the pure drug. Since ritonavir is an antiviral drug, this decrease in cell viability is expected. When the drug is encapsulated in nanoparticles, cell viability is much higher. When comparing MIP NPs and NIP NPs, cell viability was observed to be lower in MIP NPs due to the drug content. The cell viability of NIP NPs, reaching up to 80%, is the strongest evidence that the synthesized nanoparticles are non-toxic.

## 4. Limitations and Challenges

MIP NPs have gained an important place in analytical and biomedical applications thanks to their advantages, such as high selectivity, stability, and cost-effectiveness. However, several critical challenges need to be overcome to expand their applicability. In these applications, homogeneous binding sites are desired for accurate and stable performance. Conversely, heterogeneous binding sites lead to non-specific interactions and reduced binding capacity. Parameters such as mixing, temperature control, and monomer-to-crosslinker ratios cannot be maintained with the same efficiency in large reactors. This leads to problems such as batch-to-batch variability and heterogeneity in binding sites. Achieving a high imprinting factor and specificity is crucial for selective recognition in complex matrices containing structurally similar molecules [231]. Another problem occurs when the target molecule cannot be effectively removed from the polymer, which significantly affects the performance of MIP NPs. This led to significant problems such as errors in results, reduced accuracy, precision, and reusability. In DDS, this leads to decreased therapeutic efficacy and treatment success. Another problem associated with the target molecule is its sudden leakage, which may present a toxicological risk in DDS applications. In sensor and extraction systems, this causes a decrease in binding efficiency [232]. The use of organic solvents facilitates and ensures the formation of non-covalent interactions between the functional monomer and the target molecule. Any residual organic solvents remaining in the polymer can cause toxicity or cell death in healthy cells. Furthermore, the use of these solvents contradicts the principles of green chemistry. In addition, drug release performance in aqueous environments may differ significantly [24]. Regarding the monomers used in polymer synthesis, methacrylic acid derivatives such as HEMA, MAA, and MBA have suitable biocompatibility and an acceptable toxicity profile. In DDS applications, efforts are being made to reduce the potential cytotoxicity of monomers and increase biodegradability through the integration of biocompatible and biodegradable materials such as PLGA, lactic acid, cyclodextrin, and chitosan. However, since reusable and durable recognition sites are needed in sensors and extraction systems, biodegradable materials are not preferred.

Although MIP NPs have shown promising results at the laboratory level, their transition to industrial-scale production remains limited. The transition from laboratory scale to industrial scale has not been achieved except for a few companies [233].

One of the most critical challenges associated with MIP NPs in DDS is the limited data available on their biocompatibility, toxicity, and long-term safety. To ensure biodegradability, biocompatible molecules are generally preferred in DDS applications. Most studies have focused solely on laboratory-scale drug loading and release experiments. Nevertheless, critical challenges such as improving biocompatibility, reducing cytotoxicity to healthy cells, enhancing biodegradability, and ensuring long-term safety need to be addressed. Besides, important issues such as intracellular interactions, immune responses, potential toxic byproducts, and long-term accumulation within the body have not been adequately studied. These shortcomings represent a major barrier to the clinical application of MIP NPs. Although the challenges are significant, many researchers are trying to overcome these challenges in this field due to the advantages of MIP technology.

## 5. Conclusions and Future Perspectives

MIPs are effectively used in a wide range of areas such as sample preparation, sensing, separation, controlled release, catalysis, and imaging. Although sample preparation techniques have been studied for many years, they still require further development. MIP NPs selectively concentrate the target analytes, allowing the analysis of complex samples with high accuracy and sensitivity. The studies reported in sample preparation applications demonstrate that MIP nanoparticle sorbents can effectively preconcentrate drugs in various complex matrices with high specificity and selectivity. MIP NPs are also highly effective in increasing adsorption capacity and efficiency. As green approaches have recently gained importance, their use in the synthesis of MIP sorbents is expected to increase. It is anticipated that growing efforts will focus on the development of faster, fully automated, and intelligent platforms directly integrated with detection methods. Moreover, the development of durable, reusable, and compact MIP NP sorbents will minimize waste generation, reduce time and energy consumption, and enable portable and on-site analytical platforms. The preparation of adsorbents that maintain their selectivity and adsorption capacity over long extraction cycles will also be crucial.

Additionally, MIP NPs can be combined with electrochemical and optical platforms to produce selective and sensitive detection tools in sensor systems. The reported examples demonstrated in this review showed that the high surface area provided by the nanosizes significantly increases the sensitivity and selectivity of the developed nanosensor and provides a great detection response for drugs in various fields, including drug quality control, food safety, and environmental monitoring. The integration of MIP sensors with microfluidic systems as well as portable and wearable devices has significantly enhanced their application potential. The combination of MIP sensors with smartphone-based platforms enables real-time, on-site, and highly selective detection of target analytes, eliminating the need for complex laboratory infrastructure. This approach offers significant potential for advancing clinical diagnostics, environmental monitoring, and food safety applications.

In drug delivery, biocompatible and stimuli-responsive MIP NPs serve as promising carriers for targeted and controlled release. Advances in synthesis methods have enabled the development of biocompatible and biodegradable polymers, which have significantly contributed to overcoming limitations such as toxicity by increasing the biomedical compatibility of MIP NPs. Despite all these developments, studies on long-term biosafety and regulatory approval processes are required for MIP NPs to be adopted in clinical applications. It should be reiterated that further research is necessary to assess their long-term safety, toxicity, and biocompatibility. Thanks to interdisciplinary collaborations and innovations in molecular imprinting technology, MIP NPs will be used more effectively in pharmaceutical sciences in the future.

## Figures and Tables

**Table 1:** Various applications of MIP NPs-based extraction techniques for the selective extraction of different drugs.

Target Drug/Property	Extraction Material	Extraction Technique	Analytical Method	Linear Range	Matrix	Recovery (%)	Ref.
Cefaclor and cefalexin/Antibiotics	Fe_3_O_4_@c-CNTs@MIP	SBSE	HPLC-DAD	20–320 ng/mL for cefaclor, 15–240 ng/mL for cefalexin	Lake water	86.5–98.6	[94]
Phenytoin sodium/Antiepileptic drug	Fe_3_O_4_@MIL-101(Cr) nanoparticles	MSPE	HPLC-UV	0.05–40 µg/mL	Plasma	89.2–94.3	[95]
Tramadol/Opioid analgesic	Fe_3_O_4_@SiO_2_@MIP	MSPE	CE capillary electrophoresis	100–3000 ng/mL	Plasma	Close to 100	[96]
Sunitinib/Cancer drug	Magnetic molecularly imprinted nanofibers	MSPE	Fluorescence spectroscopy	0.01–15.0 mg/L	Serum and capsule	94.0–99.0	[97]
Oxycodone/Narcotic analgesic	MIP@MGO/CDs NPs	UA-DSPME	HPLC-UV	1–2000 ng/mL	Urine	92.5–103.20	[98]
Valsartan/Angiotensin II receptor blocker	Fe_3_O_4_@SiO_2_-MPS@MIP	DMSPME	UV-Vis	10–100 µg/L	Plasma and urine	100.83–102.02	[99]
Atorvastatin/Statins	Fe_3_O_4_@MIP nanoparticles	VA-DMSPME	HPLC-UV	0.5–900 ng/mL	Urine	90.50–97.70	[100]
Sulfathiazole/Veterinary drugs	Fe_3_O_4_@MIP nanoparticles	SS-MSPME	HPLC-UV	5–400 ng/mL	Milk	90.74–94.11	[101]
Capecitabine/Cancer drug	pH-sensitive magnetic MIP@chitosan nanoparticles	UA-DMSPME	HPLC-UV	5–2000 ng/mL	Plasma	93.41–102.50	[102]
Quinolones, Tetracyclines, and sulfonamides/Antibiotics	Deep eutectic solvents@UMCM-1MOF/MIP	SPME	HPLC-UV	5.0–1400 µg/L	Meat and dairy products	95.1–100.0	[103]
Fluoxetine/Antidepressant drug	Magnetic chitosan/graphene oxide@MIP	MSPE	Fiber optic linear array spectrophotometry	0.8–10.0 µg/L	Water, urine	95.7–104.0	[104]
Oxytetracycline/Antibiotic	Fe_3_O_4_@SiO_2_@Thermosensitive MIP	MSPE	Fluorescence spectrometry	0.2–60 µg/L	Ice Tea, Jasmine Tea, and Green Tea	94.2–104.9	[105]
Sulfamethoxazole/Antibiotic	Fe_3_O_4_/MIP	UA-DMSPME	HPLC-UV	7–900 ng/mL	River, Spring, and Tap Waters	94.2–98.2	[106]

**Table 2:** Comparison of analytical performances of MIP-based optical sensors prepared for the selective determination of drugs.

Target	Material	Linear Range	LOD	Detection	Ref.
Chloramphenicol	Fe_3_O_4_@MIP	0.0125–0.1 nM	0.004 nM	SERS	[128]
Ciprofloxacin	ZnFe_2_O_4_@SiO_2_@Au-MIPs Nanocomposites	10^−9^–10^−4^ M	10^−9^ M	SERS	[129]
Ofloxacin	Carbon Quantum Dots/Eu^3+^@MIP	0.83–40 nM	0.25 nM	Fluorescence	[130]
Amoxicillin	N/S co-doped CDs@MIP	5.8–200 ng/mL	1.17 ng/mL	Fluorescence	[131]
Quercetin	Fe_3_O_4_@GQDs/MIP	5–220 ng/mL	0.54 ng/mL	Fluorescence	[132]
Amoxicillin	SPR nanosensor	0.1–200 ng/mL	0.0009 ng/mL	SPR	[133]
Methamphetamine	AuNPs@MIP	1.67–167.5 µM	0.66 µM	SPR	[134]
Cocaine	Polymeric nanofilm	0.2–100 µg/mL	0.1 µg/mL	SPR	[135]
Benzylpenicillin	MIP-GO SPR	1–100 ng/mL	0.021 ng/mL	SPR	[136]
Etoposide	Ag@AuNPs@hexagonal boron ni- tride@MIP	1.70 × 10^−12^–1.70 × 10^−9^ M	4.25 × 10^−13^ M	SPR	[137]

**Table 3:** Comparison of analytical performances of MIP-based electrochemical sensors prepared for the selective determination of drugs.

Target	Material	Linear Range	LOD	Detection	Ref.
Losartan	rGO@AA@MIP paper-based sensor	8.5 × 10^−7^–6.9 × 10^−2^ M	2.7 × 10^−7^ M	Potentiometry	[159]
Amiodarone	Fe_3_O_4_@PAN@MIPs	0.3–5 µg/mL for blood and 2–10 µg/mL for plasma	0.01 µg/mL for blood and 0.4 µg/mL for plasma	CV	[160]
Fluoxetine	ZnO-MAPA@MIP/GCE	1.0 × 10^−11^–1.0 × 10^−10^ M	2.67 × 10^−12^ M	DPV	[161]
Lurasidone hydrochloride	PVC/VP/MIP/MWCNTs/SPEd	10^−4^–10^−8^ M	10 nM	Potentiometry	[162]
Chlorpromazine	MIP/pTHi/Ni-MOF/Fe- MOF-5/AuNPs/GCE	0.001–900 μM	0.025 µM	SWV	[163]
Ruxolitinib	GCE/MIP@PHEMA-ThyM	0.01–0.1 pM	0.00191 pM	DPV	[164]
Dasatinib	MATyr-CNF@MIP/GCE	1.0 × 10^−14^–1.0 × 10^−13^ M	1.76 × 10^−15^	DPV	[165]
Bortezomib	CuNFs/AMPS/MIP-GCE	0.25–2.5 pM	29 fM	DPV	[166]
Daclatasvir	AA/MIP/AgNPs/GO/CPE	10^−8^–10^−2^ M	6.9 nM	Potentiometry	[167]
Favipiravir	PCD-MIP/PGE	5.0 × 10^−6^–5.0 × 10^−5^ M and 7.0 × 10^−5^–1.0 × 10^−3^ M	1.67 µM	DPV	[168]
Tenofovir	2-AP/MIP/Pt@g-C_3_N_4_/F-MWCNTs/SPEd	0.005–0.69 µM	0.0030 µM	DPV	[169]
Umifenovir	B_3_N_3_/BuMA@MIP/GCE	0.50–7.50 pM for DPV, and 0.25–5.00 pM for EIS	48.20 fM for DPV, and 23.40 fM for EIS	DPV and EIS	[170]
Alfuzosin and Solifenacin	MIP/nanoPANI/CPE	10^−6^–10^−2^ M for alfuzosin, 10^−7^–10^−2^ M for solifenacin	7.9 × 10^−7^ M for alfuzosin, 8.9 × 10^−8^ M for solifenacin	Potentiometry	[171]
Erythromycin	SPEd/f MWCNTs/PANI/MIP/ERY-ISE	4.6 × 10^−6^–1.0 × 10^−3^ M	9.6 × 10^−7^ M	Potentiometry	[172]
Cefdinir	4-ABA@ZnONPs/MIP/GCE	7.5–100 pM	2.06 pM	EIS	[173]
Norfloxacin	MIP/PEDOT/GCE	2 nM–21.11 µM	173 pM	DPV	[174]
Ofloxacin	UiO-66-NH_2_/CNTs-2@o-PD/MIP/ITO	0.1 nM–20 nM	0.03 nM	CV	[175]
Cocaine	MIP NPs@AAm	10^−9^–10^−3^ M	-	Potentiometry	[176]
3,4-methylenedioxypyrovalerone	Dopamine@MIP@MnInPSeS QDs/SPCE	6 nM–600 μM	0.28 nM	DPV	[177]

**Table 4:** Summary of some MIP NPs-based drug delivery systems.

Template	Monomer, Crosslinker, Initiator	Loading Efficiency	Release Efficiency	Cell Viability	Ref
Olanzapine	MPS, Methacrylated fructose, AIBN	89.0%	-, 216 h, pH 4.0	More than 90%, 5 days	[191]
Sodium thiopental	MAA, EGDMA, AIBN	393.8 ± 1.328%	57.7%, 78 h, pH 7.4	-	[200]
Irinotecan	2-N,2-N-diethyl-6-prop-1-en-2-yl-1,3,5-triazine-2,4-diamine, methacrylated fructose, AIBN	95 mg/g	83.2%, 7 days, pH 5.2	More than 90%, 72 h	[208]
5-Fluorouracil	AA, Glutaraldehyde, APS	89.3%	More than 40%, 48 h	-	[214]
Doxorubicin	Methacrylamide, MBA, APS	51.2%	More than 80%, 70 h, pH 5	More than 85%, 48 h	[215]
Sunitinib	MAA, EGDMA, AIBN	87.2%	79%, 24 h, pH 7.4	-	[216]

## Data Availability

Not applicable.

## Abbreviations

The following abbreviations are used in this manuscript:

2-AP	2-aminophenol
AA	Acrylic acid
Aam	Acrylamide
4-ABA	4-aminobenzoic acid
ABDV	2,2′-Azobis(2,4-dimethylvaleronitrile)
AI	Artificial intelligence
AFM	Atomic force microscopy
AIBN	Azobisisobutyronitrile
AMPS	2-Acrylamido-2-methyl-1-propanesulfonic acid
APTES	3-aminopropyltriethoxysilane
APS	Ammonium persulfate
APTMS	3-aminopropyltrimethoxysilane
BET	Brunauer-Emmett-Teller
BuMA	Butyl methacrylate
CDs	Carbon Dots
CDDS	Controlled drug delivery system
CNTs	Carbon nanotubes
COF	Covalent organic framework
CPE	Carbon paste electrode
C-SPEd	Carbon screen-printed electrode
CV	Cyclic voltammetry
DDS	Drug delivery system
DESs	Deep eutectic solvents
DFT	Density functional theory
DMSPME	Dispersed magnetic solid phase microextraction
DPASV	Differential pulse anodic stripping voltammetry
DSPME	Dispersive solid-phase microextraction
DPV	Differential pulse voltammetry
DVB	Divinylbenzene
EDOT	3,4-ethylenedioxythiophene
EDX	Energy dispersive X-ray spectroscopy
EGDMA	Ethylene glycol dimethacrylate
EIS	Electrochemical impedance spectroscopy
HEMA	2-hydroxyethyl methacrylate
HF	Hartree-Fock
FDA	Food and Drug Administration
FT-IR	Fourier transform infrared
GCE	Glassy carbon electrode
GO	Graphene oxide
GQDs	Graphene quantum dots
HEMA	2-hydroxyethyl methacrylate
HIV	Human immunodeficiency virus
HPLC	High-performance liquid chromatography
ILs	Ionic liquids
ISEs	Ion-selective electrodes
IT-SPME	In-tube solid-phase microextraction
LC-MS	Liquid chromatography mass spectrometry
L-DOPA	(S)-2-amino-3-(3,4-dihydroxyphenyl) propanoic acid
LOD	Limit of detection
MAA	Methacrylic acid
MAPA	Methacryloyl-L-phenylalanine
MATyr	N-Methacryloyl-L-tyrosine
MBA	N, N′-methylene bisacrylamide
MIP	Molecularly imprinted polymer
ML	Machine learning
MISPE	Molecularly imprinted solid phase extraction
N-Aasp	N-acryloyl derivative of aspartic acid
MM	Molecular mechanics
MOF	Metal-organic framework
MPC	Magnetic porous cellulose
MPDE	4-Methylphenyl dicyclohexyl ethylene
MPS	3-(methacryloxy) propyl trimethoxy silane
MPTMS	3-(mercaptopropyl)trimethoxysilane
MSPE	Magnetic solid phase extraction
MSPME	Magnetic solid-phase microextraction
MWCNT	Multi-walled carbon nanotube
MTX	Methotrexate
NIP	Non-imprinted polymer
NSAIDs	Non-steroidal anti-inflammatory drugs
o-PD	o-phenylenediamine
p-APBA	p-aminophenyl boronic acid
PAN	Polyacrylonitrile
PANI	Polyaniline
PCD	Poly-carbidopa
PEC	Photoelectrochemical
PEDOT	Poly(3,4-ethylenedioxythiophene)
PGE	Pencil graphite electrode
Ppy	Polypyrrole
PS-DVB	Poly(styrene-divinylbenzene)
PT	Polythiophene
pTHi	Polythionine
PVC	Poly(vinyl chloride)
rGO	Reduced graphene oxide
RSD	Relative standard deviation
SBSE	Stir bar sorptive extraction
SEM	Scanning Electron Microscopy
SERS	Surface-enhanced Raman Scattering
SPCE	Screen-printed carbon electrode
SPE	Solid-phase extraction
SPEd	Screen-printed electrode
SPME	Solid-phase microextraction
SPR	Surface plasmon resonance
SS-MSPME	Syringe-to-syringe magnetic solid-phase microextraction
SWV	Square wave voltammetry
TDM	Therapeutic drug monitoring
TEOS	Tetraethyl orthosilicate
TFME	Thin-film microextraction
ThyM	Thymine methacrylate
UA-DMSPME	Ultrasonic-assisted Dispersed magnetic solid phase microextraction
VA-DMSPME	Vortex-assisted-Dispersed magnetic solid phase microextraction
VP	1-vinyl-2-pyrrolidine
VTES	3-vinyltriethoxysilane
VTMS	vinyltrimethoxysilane
WADA	World Anti-Doping Agency
XRD	X-ray diffraction
Zn NFs	Zinc nanoflowers
